# Co-culturing of Fungal Strains Against *Botrytis cinerea* as a Model for the Induction of Chemical Diversity and Therapeutic Agents

**DOI:** 10.3389/fmicb.2017.00649

**Published:** 2017-04-19

**Authors:** Rachel Serrano, Víctor González-Menéndez, Lorena Rodríguez, Jesús Martín, José R. Tormo, Olga Genilloud

**Affiliations:** Fundación MEDINA, Centro de Excelencia en Investigación de Medicamentos Innovadores en AndalucíaGranada, Spain

**Keywords:** co-culturing, fungal interactions, phytopathogens, antifungal agents

## Abstract

New fungal SMs (SMs) have been successfully described to be produced by means of *in vitro*-simulated microbial community interactions. Co-culturing of fungi has proved to be an efficient way to induce cell–cell interactions that can promote the activation of cryptic pathways, frequently silent when the strains are grown in laboratory conditions. Filamentous fungi represent one of the most diverse microbial groups known to produce bioactive natural products. Triggering the production of novel antifungal compounds in fungi could respond to the current needs to fight health compromising pathogens and provide new therapeutic solutions. In this study, we have selected the fungus *Botrytis cinerea* as a model to establish microbial interactions with a large set of fungal strains related to ecosystems where they can coexist with this phytopathogen, and to generate a collection of extracts, obtained from their antagonic microbial interactions and potentially containing new bioactive compounds. The antifungal specificity of the extracts containing compounds induced after *B. cinerea* interaction was determined against two human fungal pathogens (*Candida albicans* and *Aspergillus fumigatus*) and three phytopathogens (*Colletotrichum acutatum*, *Fusarium proliferatum*, and *Magnaporthe grisea*). In addition, their cytotoxicity was also evaluated against the human hepatocellular carcinoma cell line (HepG2). We have identified by LC-MS the production of a wide variety of known compounds induced from these fungal interactions, as well as novel molecules that support the potential of this approach to generate new chemical diversity and possible new therapeutic agents.

## Introduction

The continued emergence of new resistances to the existing drugs, even for the most common organisms, is challenging the future use of the limited current therapies. Invasive fungal infections are an important cause of morbidity and mortality, especially in immunocompromised patients, and the limited therapeutic options require new approaches to replenish the drug discovery pipeline with novel potential drug candidates that might respond to these unmet clinical needs (Roemer and Krysan, 2014). Microbial natural products represent one of the most important resources for the discovery of novel drugs (Koehn and Carter, 2005). So far more than 42% of known bioactive compounds have been described as produced by filamentous fungi and many of these molecules with pharmacological applications were developed to the clinic and have been broadly used as antibiotics and antifungals among other applications (Demain, 2014; Schueffler and Anke, 2014). Despite the capacity to produce a broad diversity of compounds, most orders of filamentous fungi still remain poorly explored and are potential sources of novel molecules with applications not only in human health, but as well in agriculture and other industrial sectors (Bacon and White, 2000; Strobel and Daisy, 2003; Kusari et al., 2012). Furthermore, mining of fungal genomes has revealed the high number of gene clusters involved in the biosynthesis of fungal SMs (SMs) that are not detected in strains cultivated in laboratory conditions and many approaches have been proposed in the recent years to (Brakhage et al., 2008; Rank et al., 2010).

SMs have been proposed to be produced as defense mechanisms in the microbial environment (Davies, 1990), but recent hypothesis suggest their role as chemical signals in microbial cell communication between organisms of the same and distinct species as well as with host cells (Partida-Martinez and Hertweck, 2005). Bacteria and fungi are in constant interaction in their natural environment, living in close contact and competing for resources. Microbial interactions with different combinations of microorganisms have been extensively reported and have shown how they can modulate fungal metabolism and to induce the production of new bioactive molecules (Brizuela et al., 1998; Brakhage et al., 2008; Combès et al., 2012). These interactions can lead to the activation of complex regulatory mechanisms, and finally unlock the biosynthesis of a broad diversity of natural products (Bertrand et al., 2014).

Co-cultures have proved to be an effective tool to simulate the physiological conditions that occur during the interactions of fungi in their natural environment and may have an enormous potential for the discovery of new molecules with industrial applications (Bader et al., 2010; Brakhage, 2013; Netzker et al., 2015). Whereas traditional screening processes involve culturing a single microbial strain to discover new bioactive compounds, the use of co-cultures present new opportunities for the activation of cryptic biosynthetic pathways. Microorganisms can sense the presence of other microorganisms, triggering a general response derived from this antagonistic reaction that promotes changes in the morphology of both microorganisms in the interaction zone and the induction of SMs, enzymes, and other compounds in the area of communication (Hynes et al., 2007).

Such culturing conditions have facilitated the detection of compounds which are not produced when individual fungi are cultured alone (Hynes et al., 2007). Analytical methods have permitted to detect changes in the metabolite profiles that vary depending on the interacting fungi (Peiris et al., 2008; Rodrıguez-Estrada et al., 2011). Different co-culturing techniques have been developed for this purpose including liquid and solid media, but all approaches consist on culturing two or more microorganisms in a single confined environment to facilitate interactions and induce further chemical diversity (Bertrand et al., 2013). Some examples of compounds derived from these interactions include long-distance growth inhibitors reported between *Trichophyton rubrum* and *Bionectria ochroleuca* (Bertrand et al., 2013), the production of acremostatins A–C, by *Acremonium* sp. when cultivated in mixed culture with *Mycogone rosea* (Degenkolb et al., 2002), the production of aspergicin, by a co-culture of two *Aspergillus* species (Zhu et al., 2011) or the production of cyclo-(l-leucyl-*trans*-4-hydroxy-l-prolyl-d-leucyl-*trans*-4-hydroxy-l-proline), when the two mangrove strains of *Phomopsis* sp. and *Alternaria* sp. are co-cultured in liquid media (Li et al., 2014). Additional studies have also reported the increase of metabolite production and the induction of new SMs during microbial interactions such as the variation of the metabolite expression detected by LC-MS in the interaction between the phytopathogen *Fusarium verticillioides* and the endophyte *Ustilago maydis* (Rodrıguez-Estrada et al., 2011), or the induction of 12 metabolites by *Paraconiothyrium variable* when co-cultured with *F. oxysporum* (Combès et al., 2012).

Phytopathogen fungi are a wide and phylogenetically diverse group of microorganisms infecting plants or even causing serious plant diseases (Heydari and Pessarakli, 2010). *Botrytis cinerea* is one of the most important phytopathogen fungi according to the economic impact of the damages produced by its high infection rates (Dean et al., 2012). *B. cinerea* is the causing agent of the gray mold in a wide number of plants during all production cycle, including their storage and transport (Couderchet, 2003; Soylu et al., 2010; Dean et al., 2012; Wang et al., 2012). Furthermore, *B. cinerea* is an excellent model for the study of fungal infection processes given its polyphagic and necrotrophic characteristics. This fungus promotes a rapid destruction of the tissues of the host plant by using a broad range of pathogenic factors (lytic enzymes, activated oxygen forms, toxins or plant hormones). Access to its genome and transcriptomic analyses have identified many genes and functions involved in the infectious process (Fillinger and Elad, 2016). As a phytopathogenic fungus, *B. cinerea* is a natural competitor for many fungal strains isolated from plants. Therefore, we propose in this study the use of fungal co-cultures with *B. cinerea* to challenge and activate cryptic pathways in fungal strains isolated from diverse plant environments and to identify potential producers of new antifungals. Chemical dereplication of known antifungals and an initial characterization of induced activities against a panel of human and plant fungal pathogens was also carried out to perform an extensive evaluation of this model interaction in the discovery of new antifungal agents.

## Materials and Methods

### Fungal Strains

Fungal strains used in this work were obtained from Fundación MEDINA Culture Collection. The 762 wild-type strains were grown on Petri dishes of 55 mm of diameter with 10 mL of YM medium (yeast extract Difco^TM^ 1 g, malt extract Difco^TM^ 10 g, agar 20 g, and 1000 mL deionized H_2_O), and incubated in darkness for 10–14 days at 22°C and 70% relative humidity (RH). Strains were selected from different environments, to ensure a broad and representative fungal community from soils, leaf litters, plant endophytes and epiphytes, and rhizosphere isolates from different geographical origins and environments.

Four phytopathogenic fungi were also used: *B. cinerea* CBS 102414, *Colletotrichum acutatum* CF-137177, *F. proliferatum* CBS 115.97 and *Magnaporthe grisea* CF-105765.

Human pathogens used in the agar based assays include *Aspergillus fumigatus* ATCC 46645 and *Candida albicans* MY 1055.

MEDINA fungal Collection strains were identified according to their morphological characters, the ITS1-5.8S-ITS2 region and the first 600 nt of the 28S gene of each strain were sequenced and compared with GenBank^®^ or the NITE Biological Resource Center^1^ databases by using the BLAST^®^ application.

### *B. cinerea* Co-culturing Induction on Agar

Fungal strains were confronted against *B. cinerea* using co-culturing methods on agar. *B. cinerea* strain that was grown in 250 mL Erlenmeyer flasks containing 50 mL of SMYA medium (neopeptone Difco^TM^ 10 g, maltose Fisher^TM^ 40 g, yeast extract Difco^TM^ 10 g, agar 4 g, and 1000 mL deionized H_2_O), and incubated at 220 rpm, 22°C and 70% RH for 3 days. Co-culture Petri dishes of 55 mm with 10 mL of 2% malt agar (malt extract Difco^TM^ 20 g, agar 20 g, and 1000 mL deionized H_2_O), were inoculated with 0.2 mL of *B. cinerea* liquid culture on one side of the plate, and an agar plug of the test strain to be induced was placed on the opposite site of the plate. All Petri dishes were incubated at 22°C and 70% RH in darkness for 10 days. In parallel, axenic strains were inoculated using the same methodology.

### Generation of Extracts from Agar Zones of Positive Interactions

Extracts were prepared from two areas of the inhibition zones formed in the co-culturing plates: (a) the growth inhibition area established between the growth of both fungal strains, and (b) the edge of *B. cinerea* inhibited mycelium. Similar agar areas were extracted from the axenic controls. Agar blocks were cut using a sterilized scalpel, and extracted with acetone (3 mL) in two Falcon tubes. Falcon tubes were centrifuged at 5000 rpm for 5 min and supernatants were collected. The extraction process was repeated twice for ensuring an efficient extraction and supernatants were combined. The extracts were concentrated to dryness under heated nitrogen stream. Hundred microliter of DMSO and 400 μL of H_2_O were added sequentially to reconstitute the samples, that were transferred to HTS 96-well AB-gene^®^ plates with an automatic liquid handler Multiprobe II^®^ robot. Extracts were filtered in 96 format with 0.22 μm MultiScreen^®^ filter plates by 2000 rpm centrifugation for 5 min. Finally, the plates were heat-sealed and stored at 4°C until tested.

### *In Vitro* Antimicrobial Assays Against Fungal Pathogens

#### Agar-Based Assay for Antifungal Evaluation

Media used for agar-based antifungal assays were SDA (sabouraud dextrose agar Difco^TM^ 65 g and 1000 mL H_2_O), Malt (malt extract Difco^TM^ 20 g, agar 20 g, and 1000 mL deionized H_2_O), YM (malt extract Difco^TM^ 10 g, yeast extract Difco^TM^ 2 g, agar 20 g, 1000 mL deionized H_2_O). Depending on the phytopathogen fungus used, conidia concentration was also adjusted in a range from 1 × 10^4^ to 5 × 10^7^ conidia/mL (Moreira et al., 2014; Li et al., 2015; Ong and Ali, 2015) and plates were prepared in a temperature range between 30 and 45°C of the media for microbial damage. Twenty-five microliter of agar media containing conidia or spores were dispensed in OmniTray^®^ of 86 mm× 128 mm assay plates. Once solidified, samples were dispensed on top of the plates (10 μL) using an automated liquid handler (BiomekFX^TM^, Beckman Coulter^®^). Five antifungal agents were evaluated as positive controls, in order to determine the optimal concentrations for a standard curve for each phytopathogenic strain from 5 mg/mL to 0.005 mg/mL: amphotericin B, cycloheximide, fluconazole, itraconazole, and voriconazole. Results (not shown) indicated amphotericin B as the best standard for reference.

The assay plates were incubated in dark at 25°C and 70% RH during 24 h. The diameter and turbidity of the inhibition halos were measured to assess the antimicrobial activity using a proprietary Image Analyzer^®^ software and a stereoscope microscope (Leica^TM^ MZ16). In the first column, each assay plate contained alternated, four positive control points corresponding to amphotericin B 1 mg/mL, and four DMSO 20% negative controls. The last column contained a specific standard curve of amphotericin B, different for each pathogen. Extracts were evaluated per duplicate for each pathogen.

#### *Aspergillus fumigatus* Agar-based Assay

The *A. fumigatus* ATCC 46645 stock conidial suspension was prepared in Petri dishes of PDA and incubated at 37°C during 48 h. The plate surface was scraped with Tween 80 solution 0.1% (v/v) and a sterile loop to obtain a conidia suspension that was filtered with a sterile gauze. The conidia suspension was inoculated at a final concentration of 1⋅10^5^ spores/mL in yeast nitrogen base-dextrose agar medium, in OmniTray^®^ assay plates of 86 mm× 128 mm. Amphotericin B 1 mg/mL and DMSO 20% were used as positive and negative controls, respectively. Concentrations for the standard curve of amphotericin B were: 0.06 mg/mL–0.04 mg/mL–0.02 mg/mL–0.01 mg/mL.

#### *Candida albicans* Agar-based Assay

Frozen stocks of *C. albicans* MY1055 were used to inoculate Sabouraud Dextrose Agar (SDA) plates and were incubated for 24 h, at 37°C. Grown colonies were suspended in Sabouraud Dextrose Broth (SDB) and incubated at 37°C and 250 rpm during 18–20 h. The OD660 was adjusted to 0.4 using SDB as diluent and blank. It was used 30 mL of this overnight inoculum to inoculate 1 L of yeast nitrogen base-dextrose agar medium (YNB 67 g/L, Dextrose 10 g/L, Agar 15 g/L), dispensing in assay plates OmniTray^®^ of 86 mm× 128 mm (de la Cruz et al., 2012). Amphotericin B 1 mg/mL and DMSO 20% were used as positive and negative controls, respectively. Concentrations for standard curve finally was determined by four points of amphotericin B: 0.06 mg/mL–0.04 mg/mL–0.02 mg/mL–0.01 mg/mL.

#### Generation of Conidia and Spores for Agar-based Assays

The evaluation against the three phytopathogens *Colletotrichum acutaum*, *Fusarium proliferatum*, *and Magnaporthe grisea*, required an optimization of the assay and in each case to define the optimum method for obtaining conidia to be used in the assay.

For the sporulation of the phytopathogen fungi six different solid media were tested: CMD (cornmeal agar Microkit^TM^ 17 g, glucose Sigma–Aldrich^TM^ 1 g, agar 4 g, and 1000 mL deionized water), MALT 2% (malt extract Difco^TM^ 20 g, agar 20 g, and 1000 mL deionized water), OAT-MEAL (oatmeal 60 g, agar 20 g, and 1000 mL deionized water), PDA (potato dextrose agar Difco^TM^ 20 g and 1000 mL deionized water), SNA (KH_2_PO_4_ Merck^TM^ 1 g, KNO_3_ Merck^TM^ 1 g, MgSO_4_⋅7H_2_O Merck^TM^ 0.5 g, KCl Merck^TM^ 0.5 g, glucose Sigma–Aldrich^TM^ 0.2 g, sucrose Fisher^TM^ 0.2 g, agar 20 g, and 1000 mL deionized water), V8-AGAR (V8 juice 200 mL, agar 20 g, and CaCO_3_ Panreac^TM^ 3 g and 1000 mL deionized water) and YM. *C. acutatum*, *F. proliferatum*, and *M. grisea* strains were cultured in these media by putting three mycelial agar plugs on Petri dishes of 90 mm, and were incubated at 22°C and 70% RH for 21–28 days depending of the strain. After this incubation, all plates were evaluated under the stereoscope (Leica^TM^ MZ16), to identify the presence of conidia or spores. The best medium to induce their sporulation was selected in each case and the culturing was repeated until obtaining enough conidia to be used in the inhibition assays.

Two methods were evaluated for obtaining the highest quantity of conidia or spores: cultivating the fungal strain in submerged culture conditions during 24 h (overnight), or directly scraping the produced conidia or spores from the solid medium. In the first case, 50 mL of liquid medium were inoculated in 250 mL Erlenmeyer flasks with 12 agar plugs of mycelia grown in Petri dishes containing conidia and spores. Two different liquid media: SDB (sabouraud dextrose broth Difco^TM^ 30 g and 1000 mL deionized water) and SMY (neopeptone Difco^TM^ 20 g, maltose Fisher^TM^ 40 g, yeast extract Difco^TM^ 10 g and 1000 mL deionized water) were tested. Flasks were incubated at 220 rpm, 22°C and 70% RH during 24 h. In the second case, the surface of the sporulated fungus growing on solid media was scraped using a Tween 80 solution 0.1% (v/v) and a sterile loop to detach conidia or spores. Conidia were washed sequentially with sterile water to remove any remaining Tween 80. Finally, the resulting liquid culture was filtered with sterile gauzes to obtain a homogeneous solution of conidia and spores. The resulting solution of conidia and spores was counted in a Neubauer chamber 0.0025 mm^2^ and was adjusted the solution to the optimum concentration of conidia per milliliter.

#### *Colletotrichum acutatum* Agar-based Assay

The optimum method for obtaining conidia was to directly scrap a sporulated culture in medium YM after 21 days of incubation. Conidia were tested at several concentrations (conidia/mL): 1⋅10^5^, 5⋅10^5^, 1⋅10^6^, 5⋅10^6^, 1⋅10^7^, and 5⋅10^7^) and 1⋅10^7^ conidia/mL was selected as the optimum concentration for *C. acutatum*, permitting the best definition of inhibition halos. The amphotericin B standard curve included four points: 0.06 mg/mL–0.04 mg/mL–0.02 mg/mL–0.01 mg/mL.

#### *Fusarium proliferatum* Agar-based Assay

For this phytopathogen the method used to obtain a conidial suspension was to incubate the fungus overnight in liquid in SMY media, where the fungus was able to produce a large quantity of conidia and few hyphae. The different concentrations of conidia/mL were evaluated, and 1⋅10^6^ conidia/mL resulted the optimum concentration to ensure clear inhibition halos. Four concentrations of amphotericin B were used in the standard curve (0.50 mg/mL–0.10 mg/mL–0.05 mg/mL–0.01 mg/mL).

#### *Magnaporthe grisea* Agar-based Assay

To obtain a solution enriched in conidia of *M. grisea*, the fungus was grown in CMD medium to achieve sporulation and spores were directly scraped from the plate surface and the best inoculum concentration in the assay was established in 1⋅10^6^ conidia/mL. The amphotericin B standard curve contained four concentrations: 0.50 mg/mL – 0.10 mg/mL–0.05 mg/mL–0.01 mg/mL.

#### Evaluation of Cytotoxicity on HepG2 Cell Line

MTT (3-(4,5-dimethylthiazol-2-yl)-2,5-diphenyltetrazolium bromide) reduction rate is an indicator of the functional integrity of the mitochondria and used to evaluate cellular viability (Corine et al., 1998; Liu and Nair, 2010). In order to evaluate the cytotoxicity of extracts obtained from positive co-cultures and the corresponding axenic strains, the extracts were tested using the MTT assay against the HepG2 cell line (hepatocellular carcinoma, ATCC HB 8065) (de Pedro et al., 2013) after 24 h of incubation. Extracts were evaluated per duplicate.

### Chemical Characterization of Positive Microbial Interactions

In order to identify known antifungals and induced natural products, the active extracts identified from the co-culture of *B. cinerea* with the tested fungi were analyzed by LC-MS and their chemical profiles were compared to internal proprietary databases of more than 900 known active molecules. LC-MS and database matching of known SMs were based on the LC-MS analyses in the low (LR) or high resolution (HR) mode and were performed as previously described (Martín et al., 2014; Pérez-Victoria et al., 2016). Database searching was performed against the MEDINA proprietary database of microbial metabolites or the Chapman & Hall Dictionary of Natural Products (v25.1).

## Results

### Fungal Strains Confronted to *B. cinerea*

The study has evaluated the induction of new SMs from a wide population of 762 fungal strains of high taxonomic and geographic diversity when co-cultured with *B. cinerea*. The fungal strains were selected according to ecological characteristics of the environment where they were isolated, in the aim of prioritizing ecosystems where possible interactions with *B. cinerea* could be present. The strains were isolated from environmental samples collected in four climatic regions including: (a) a temperate group of 125 strains ranging from Argentina to Japan, New Zealand, the Republic of Georgia and western Europe, (b) an arid climate group of 102 strains from arid areas in Australia, Chile, Arizona (USA) and Almeria (Spain), (c) a tropical group of 282 strains selected from different tropical forests in Africa, Central and South America and Indic Ocean countries, and (d) a Mediterranean group with 253 strains from Southern Europe and South Africa.

The fungal strain population was selected from a wide diversity of habitats, identifying among them fruiting bodies (77), herbivore dung (27), leaf litter (187), lichens (18), plant rhizospheres (20), soils (148), and other plant materials (285). The largest group of strains was represented by strains isolated from leaf litter (187) and soils (148) associated to agricultural crops. Leaf litter is a unique source of saprophytic fungi decomposers of plant organic matter (Bills and Polishook, 1994; Lunghini et al., 2013) and soil fungi have an important function in the recycling of habitats of high fungal diversity (Sayer, 2006; Nihorimbere et al., 2011; Chandrashekar et al., 2014). Strains isolated from different plant rhizospheres are of special interest given their ability to generate interactions with plant roots to ensure absorption of nutrients and protection against other microbial pathogens. Finally, endophytes and epiphytes isolated from plants are natural competitors with plant phytopathogenic fungus, what can facilitate finding strains antagonistic to *B. cinerea*.

### Eliciting Positive Microbial Interactions on Agar Plates

Fungal co-culturing techniques have been used previously to mimic *in vitro* microbial interactions occurring in their natural environment and to identify new antifungal compounds (Liu et al., 2001; Kim et al., 2015; Lopes et al., 2015; Ting and Jioe, 2016). We selected *B. cinerea* as a model to be used in fungal co-cultures and to induce the production of new SMs derived from fungal interactions. *B. cinerea* is a worldwide spread fungus that can be found in most of the environments where strains were isolated. In addition, it is an invasive fungus with fast growth, which facilitates the identification of antagonisms presented by other fungi.

Fungal co-cultures on solid media were incubated for 10 days, the time required by *B. cinerea* to grow and invade the whole Petri dish. From the 762 strains studied, 93 presented a positive antagonism (12% hit rate) observing a clear inhibition of the growth of *B. cinerea*. These 93 strains were evenly distributed among the different groups of strains of the tested population, both according to climatic and ecological aspects (Results not shown), indicating that the antagonism against *B. cinerea* was uniformly distributed within our group of strains.

**FIGURE 1:**
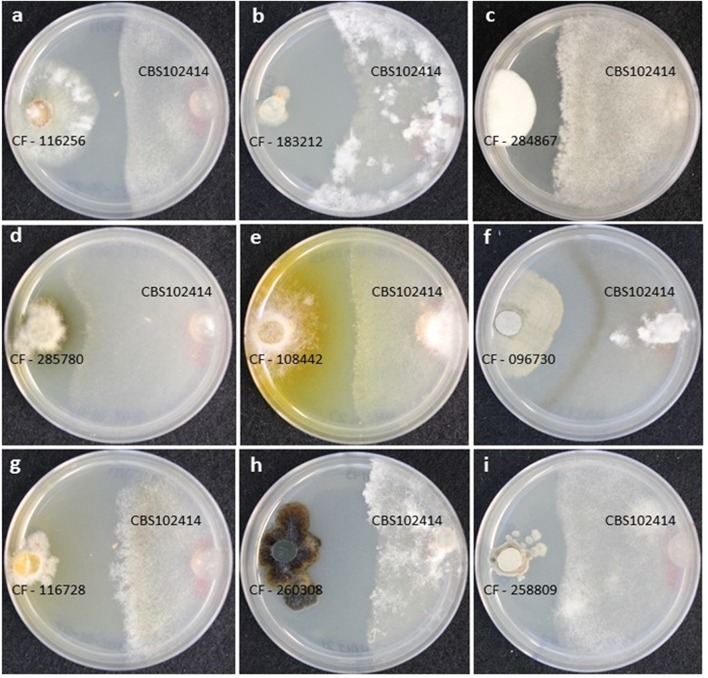
**(A–I)** Different growth morphologies and colony-front patterns in *Botrytis cinerea* CBS102414 as a response to the microbial interaction with wild-type fungal strains.

In addition, to the growth inhibition, we observed differences in the growth morphology and colony-front pattern of *B. cinerea* including: (i) a clear front formed from a homogeneous mycelium (**Figures 1a,d,e,i**); (ii) a diffused front with overgrown aerial mycelium (**Figures 1b,h**); (iii) a diffused front with an homogenous pigmented mycelium (**Figures 1c,g**), and (iv) a clear front with accumulation of pigments at the front of the inhibited mycelium (**Figure 1f**). Typically, it is possible to identify SMs that diffused in the inhibition zone from the induced strains and that are diffusing as a gradient across the agar. These molecules can include both signaling and reacting compounds induced as result of the interaction as well as other SMs produced constitutively by each of the strains (Schulz et al., 2015).

**FIGURE 2:**
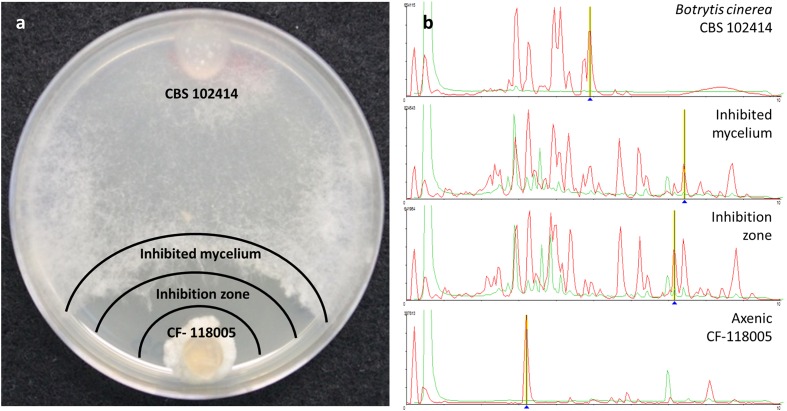
**Zones of microbial interaction evaluated (a)** for antifungal activities and LC-MS chemical profiling **(b)**

Each area of inhibition resulting from a positive interaction on the agar plate and the diffusion of chemical signals was extracted, as well as the front-colony area of the *B. cinerea* mycelium where the fungus may be responding to the challenge (**Figure 2**). This differentiation of two zones has permitted to evaluate the relative amounts of any compound contained in the extract and to indicate according to its diffusion gradient the source of the compound. With the aim of better understanding and evaluating the nature of the active molecules induced from the fungal interactions with *B. cinerea*, we screened the extracts against a panel of fungal pathogens to evaluate their antifungal specificity and performed a LC-MS chemical profiling as a first attempt to characterize the chemical composition in the extracts (**Figure 2**).

.

#### Antifungal Activities against the Plant Pathogens

We selected three different phytopathogen to test the antifungal activities contained in the zone of inhibition extracts, evaluate their antifungal spectrum and characterize their specificity. The genus *Colletotrichum* is one of the most common and important genus among the phytopathogenic fungi, due to the capability to induce the anthracnosis in fruits and vegetables (Cacciola et al., 2012; Dean et al., 2012; Gang et al., 2015). Species as *C. acutatum* and *C. gloeosporioides* are serious threats for olive crops (*Olea europea*) an important cause of large losses in this agricultural sector (Oliveira et al., 2005; Rouhma et al., 2010). *Fusarium* spp. involve a lot of plant pathogenic species, and affect a large variety of crops, ranging from corn, to wheat and other cereals, as well as the majority of fruits and even ornamental plants (Díaz et al., 2013). The third phytopathogen is *M. oryzae*, one of the most important phytopathogen given damages impact on rice (*Oryza sativa*). In addition, *M. oryzae* is the type organism used in the research on the interactions between this pathogen and its hosts plant (Dean et al., 2012). Two species of the genus *Magnaporthe* (*M. grisea* and *M. oryzae*) are the main causative agent of rice blast disease, that is considered the most destructive infection that can promote losses up to 100% of ground production (Viaud et al., 2002; Kohli et al., 2011; Koeck et al., 2012). In general, all three phytopathogenic species are very studied due to the wide range of plants they can infect, above and especially cereal crops (Kohli et al., 2011).

**FIGURE 3:**
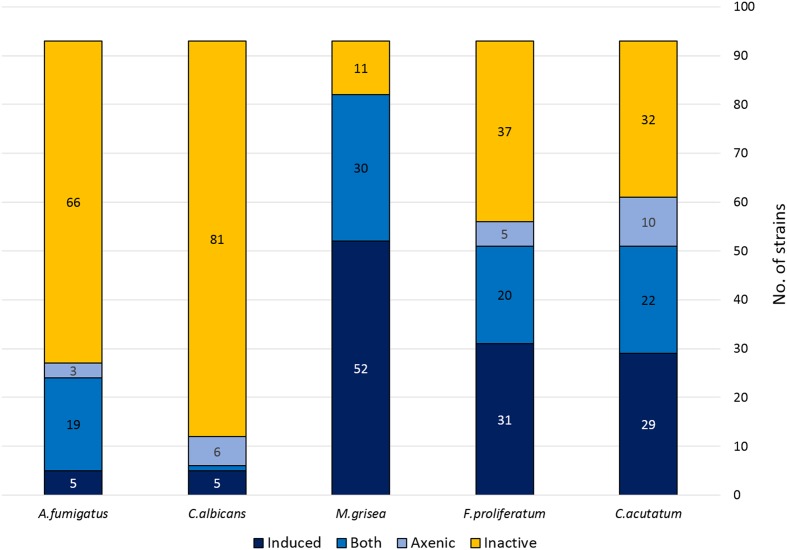
**Antifungal activity distribution of the co-culture extracts**.

Most of the co-culture extracts presented a broad antifungal spectrum against the plant pathogens. As any as 51 extracts from the 93 strains that had a positive interaction with *B. cinerea*, presented activity against *C. acutatum*. From then 29 antifungal activities (57%) were not produced by the corresponding axenic controls, and had been subsequently be induced with the co-culture. Similarly, 57 extracts were active against *F. proliferatum*, from which 31 (55%) were not present in the corresponding axenic controls. Finally, as many as 82 extracts were also active against *M. grisea* with 51 of the activities only observed in co-cultures (62%). There are also cases of strain extracts that presented antifungal activity against the phytopathogens *C. acutatum* (10 extracts), and *F. prolifetatum* (5 extracts) when cultured axenically, but lost the activity when co-cultured with *B. cinerea*, were 10 for *C. acutatum*, 5 for *F. proliferatum* and none for *M. grisea* (**Figure 3**).

### Antifungal Activity Against Human Pathogens

The active extracts were also screened against the human pathogens *Candida albicans* and *A. fumigatus*. In this case the active extracts were detected to a lesser extent that those previously observed against the phytopathogen panel. Only 6 from the 93 co-culture extracts were active against *Candida albicans*. From these, 5 of these activities (83%) were only observed in the co-culture and were not observed in the extracts prepared from their axenic controls. An opposite situation was found with another group of six strains that only produced antifungal activities against *C. albicans* when grown axenically, and that were not detected in co-culture. In the case of *A. fumigatus*, 24 extracts showed some activity against this pathogen, but only 5 of them (21%) were induced by the interaction, and only 3 were active when obtained from strains cultured axenically (**Figure 3**).

### Chemical Dereplication and Characterization of Induced Antifungals

The active extracts were dereplicated by LC-MS against our databases of known compounds (Pérez-Victoria et al., 2016), and the following antifungal molecules were identified by fingerprint matching of HPLC retention time, UV and LC-LRMS spectra data: cercosporamide, 7-chloro-6-methoxymellein, cyclosporin A, equisetin, gliotoxin, globosuxanthone, griseofulvin, mycophenolic acid, mycorrhizin, palmarumycin C15, preussomerin, thrichothecin, virescenoside C, violaceol I/II.

Among them preussomerins were the family of molecules most frequently detected in the active extracts, especially the preussomerin analogs F and B, and followed in frequency by preussomerin A. These antifungal metabolites have been described in *Sporormiaceae*, the genus *Preussia* sp. and one species of *Dothidiales* (*Hormonema dematioides*) (Quesada et al., 2004). Palmarumycin C15, closely related structurally to preussomerins and produced by species of *Pleosporales*, was the second most abundant molecule de-replicated. Other known antifungals such as equisetin (produced by several species of *Fusarium* sp.), globosuxanthone A (produced by species of *Hypocreales* and *Pleosporales*), griseofulvin (produced by several species of *Penicillium* sp.) and virescenoside C (produced by species of *Hypocreales*) (Cagnoli-Bellavita et al., 1970; Burke et al., 2005; Chooi et al., 2010; Yamazaki et al., 2012) were detected only in one or two strains.

Most of the de-replicated known antifungals were detected both in the inhibition zones and the *B. cinerea* mycelium front, being the differences in their LC-LRMS areas indicating their diffusion gradients and in consequence the producer organism. This allowed to identify if they active compounds were produced by the strain confronted to *B. cinerea* or by *B. cinerea* itself as a mechanism of defense.

Despite the presence of some known compounds the initial dereplication did not find any known antifungals in as many as 70% of the co-culture extracts. To evaluate the efficiency of the co-culturing approach with *B. cinerea* to induce chemical diversity we compared the metabolite profiles of the extracts independently of their biological activities. For this purpose, we analyzed by LC-HRMS the extracts and predicted tentative molecular formulas of their major components in order to identify the new induced compounds, disregarding constitutive metabolites (Pérez-Victoria et al., 2016).

### Cytotoxicity of Active Extracts

The extracts were tested against the human HepG2 cell line to evaluate the production of cytotoxic compounds. Regarding specificity against the plant pathogens and their cytotoxicity against the human HepG2 cell line, 4 extracts (14%) that presented activity against *C. acutatum*, and one against *F. proliferatum* (3%) were all of them as well cytotoxic; finally, from the 23 extracts with activity with *M. grisea* (45%), 7 of them were cytotoxic.

Among the compounds dereplicated in the extracts by LC-LRMS, most of them were identified as known cytotoxic agents or antibiotics. This identification was also dependent on the amounts of the cytotoxic compound in the extracts above the detection limits. For amounts of active compounds under the level of detection of LC-LRMS, we analyzed in detail the LC-HRMS profiles to check if any of those components was present in traces. A general evaluation of the number of induced SMs detected by LC-LRMS indicated that, as average the number of components produced in high titters in the extracts from positive interactions were 21.2 ± 4.8 and they were equivalent to doubling the number of metabolites observed in the extracts from the axenic control (12.2 ± 3.1). When these components were analyzed in detail by LC-HRMS and their molecular formula predicted by HRMS isotope patterns, we could identify as average 9.3 ± 2.3 metabolites produced by *B. cinerea* and present in the interactions. Clearly, the difference observed in the number of metabolites detected in the microbial interactions was due to the addition of the compounds resulting from the interaction between the confronted strains.

### LC-HRMS Profiling of Active Extracts

The chemical dereplication of the 93 positive antagonisms indicated that 70% of the co-cultured pairs presented antifungal activities that were not identified as already know natural products when compared by LCMS to our database with more than 900 standards of families of known compounds. To look for the presence of other known compounds not contained in our databases, we analyzed the LC-HRMS profiles of every extract obtained for each strain in an interaction. In general, whereas the total number of SMs was similar to the addition of those observed in the axenic cultures, in many cases the molecules were different, confirming that the microbial interactions had induced changes the secondary metabolite profiles on the confronted strains. A tailor-made analysis of each pair was clearly necessary to understand each interaction and to explain the different responses that their extracts presented when characterized for antifungal activities.

**FIGURE 4:**
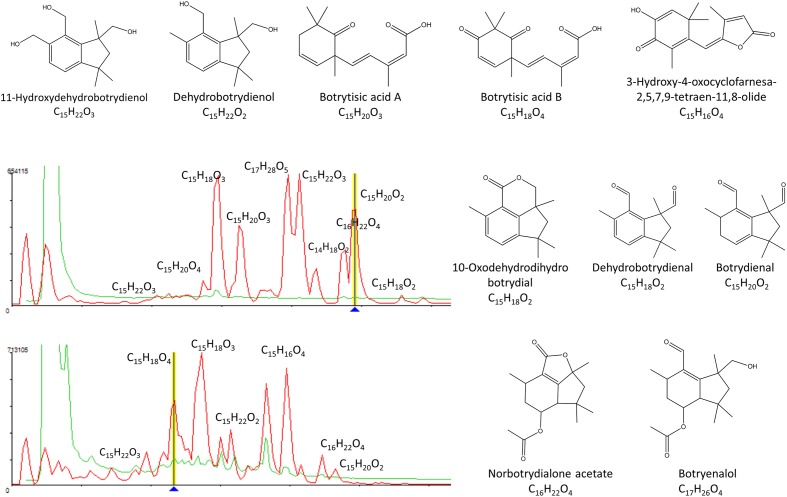
**Tentative identification of secondary metabolites induced in *B. cinerea* as a response to the microbial interactions**.

We could identify tentatively the SMs induced in *B. cinerea* as a reaction to the microbial interaction. according to the molecular formula prediction and unique matching with metabolites described from *B. cinerea* in the Chapman & Hall Dictionary of Natural Products: 11-hydroxy-dehydro-botrydienol (C_15_H_22_O_3_), botrytisic acid A (C_15_H_20_O_3_), botrytisic acid B (C_15_H_18_O_4_), dehydro-botrydienol (C_15_H_22_O_2_), 3-hydroxy-4-oxocyclofarnesa-2,5,7,9-tetraen-11,8-olide (C_15_H_16_O_4_), norbotrydialone acetate (C_16_H_22_O_4_), botrydienal phytotoxin (C_15_H_20_O_2_), 10-oxo-dehydro-dihydro-bodtrydial (C_15_H_18_O_2_), hydrated-botryenalol (C_17_H_26_O_4_⋅H_2_O), and dehydro-botrydienal (C_15_H_18_O_2_) (**Figure 4**).

The active compounds identified by LC-HRMS dereplication in as many as 25 strains corresponded to molecules contained both in the axenic and co-culture extracts. The molecules 2-(-hydroxy-5-methoxyphenoxy)acrylic acid, integrastin B and palmarumycin C15 were dereplicated among other unknown natural products produced in the axenic controls in the strains CF-185391, CF-177133, CF-192842, CF-111323, CF-200182, and CF-210766. Similarly, the strains CF-116869, CF-096730, CF-195204, produced australifungin, botryendial phytotoxin, and tentatively a molecule identified as cissetin. Other molecules such as 3-*O*-acetyl-botcineric acid, equisetin, enniantin I and G, 11,12-hydroxy-eudesm-4-en-3-one, cordyol C, illudin C3, illudinic acid, integrastin B, 7-chrolo-6-methoxy-mellein, cis-4-hydroxy-6-deoxy-scytalone or xylarine, and tentatively, citreoviridin licicolinic acid A, 4-hydroxy-5-methyl-mellein, were also dereplicated by LCMS within this group of strains.

Once identified most of the known metabolites produced by *B. cinerea*, and those produced by the other fungal strains independently of the interaction, we studied the molecules only present in the co-culturing extracts that could correspond to induced compounds produced by each of the other fungi in the microbial interaction. These compounds were more abundant in the inhibition area and in less amount or not present both in the axenic strain or in the *B. cinerea* inhibited mycelium. Upon LC-HRMS analysis and molecular formula prediction of the three extracts for each co-cultured pair of strains, we could identify some molecules that were only present in the co-culturing inhibition extract for each pair.

**FIGURE 5:**
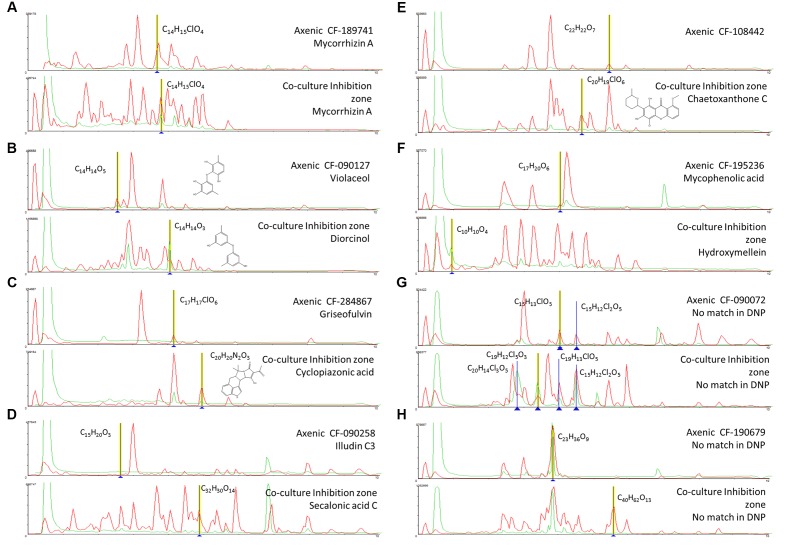
**(A-H)** Comparison of the secondary metabolite profiles produced by each axenic wild type fungal strains and its co-culturing with *B. cinerea*. Tentative LCMS identification of the main induced secondary metabolites is highlighted in each case.

One of these examples is chaetoxanthone C that was only detected in the extract from the inhibition zone of the co-culture of CF-108442 *Verticillium* sp. (**Figure 5**).

Among the strains that produced induced antifungal activities against human fungal pathogens we can highlight the extract from the co-culture with the strain CF-118005 was found to contain as many as 21 induced metabolites without considering the known compounds produced by *B. cinerea*. One of them could be tentatively identified as SNF 4794-7 against our database of known compounds. Similarly, in the strain CF-183212 we identified induced components with m/z 312.1930 (CHO). Cordyol C was induced in the strain CF-187233, along with C_15_H_16_O_4_, a component possibly related to the original C_15_H_16_O_5_ detected in its axenic culture. In the case of CF-189741, 17 components were induced in this strain including a family of 210 nm UV absorbing metabolites, including mycorrhizin A and a chlorinated C_14_H_15_ClO_4_ among them. The strain CF-246868 also produced 19 induced SMs that included palmarumycins C7 and C15 and other preussomerin UV-like spectrum components. A similar situation could be observed for CF-090071 with a family of induced UV absorbance components. Finally, CF-268787 produced penicillic acid when confronted to *B. cinerea* among other 11 induced components.

In the group of five strains that produced induced antifungal extracts with a broad spectrum against the three plant pathogens we identified very few UV absorbing components in CF-116728, chaetoxanthone C, and one chlorated compound (C_20_H_19_ClO_6)_ among other 7 components in CF-108442, palmarumycn C13 as well as other 6 components in CF-176883, and 8 and 12 induced metabolites in CF-185636 and CF-262323 respectively. In addition, two strains CF-288225 and CF-185653 presented 13 and 9 clearly induced SMs.

The strain CF-190679 with specific activity against *Fusarium*, presented multiple components (12), many of them with molecular formulas not described for any known natural products and without match in the Chapman & Hall Dictionary of Natural Products (DNPv25.1). The co-culture extracts of CF-090258 and CF-153629, with specific activity against *C. acutatum*, contained, respectively, the secalonic acid C along other 14 SMs, and several induced metabolites, some of them with molecular formulas (i.e., C_11_H_15_ClO_5_) not described previously for known fungal natural products.

Among the nine strains with induced activities specific to *M. grisea* (CF-090072, CF-090127, CF-110925, CF-118101, CF-195236, CF-209253, CF-213368, CF-284867, and CF-285353) we identified the production of diorcinol, destruxin A1/A4, antibiotic FR-49175, bis-dethio-bis-methyl-thio-gliotoxin, 3-hydroxymellein, 3,4-dihydro-3,8-dihydroxy-3-methyl-1H-2-benzopyran-1-one, pleuromutilin, cyclopiazonic acid, and a range of 7 to 19 additional SMs not present in the DNP.

## Discussion

### Distribution of the Positive Interactions Among the Population of Confronted Fungi

The distribution of the strains that presented positive antagonism against *B. cinerea* presented similar distribution the initial population of strains confronted, both in climate and ecological isolation origins, confirming that all climates and ecological source niches included in the study presented strains equally susceptible of respond to interactions with *B. cinerea*. Among them only 34% of the strains produced broad-spectrum antifungal compounds in the interaction zone showing activity against all three plant pathogens.

### Identification of Metabolites Produced in the Axenic Cultures

Two groups of toxins have been described in literature to be produced by *B. cinerea*: the sesquiterpene botryanes and the polyketides botcinins. Both may facilitate its plant infection process (Reino et al., 2004; Tani et al., 2006; Collado et al., 2007). As response to the attack of the co-culturing strain, we confirmed that *B. cinerea* produced some of these natural products that were included among the group of molecules that were detected in the LC-HRMS chemical profiles of the active extracts. These molecules were present in higher amounts in the extracts obtained at the front of the colony of the *B. cinerea* inhibited mycelium than in the inhibition area between confronted strains (as indicated by area comparison of their LC-HRMS peaks between both extracts per each co-culture analyzed).

For the remaining strains, the LC-HRMS characterization and dereplication of compounds from axenic versus co-cultures against databases of know compounds indicated that, for 25 strains, their positive antagonic interaction with *B. cinerea* could be explained by the presence of constitutive molecules also present in the corresponding axenic culture and were not induced as a reaction to the attack of the confronted strain. None of them presented inhibition of human pathogenic fungi, but they could be classified in two groups formed by nine strains with extracts with broad spectrum against the plant pathogens and 16 strains with active extracts specific to *M. grisea*. In the first group are included the strains CF-185391, CF-177133, CF-192842, CF-111323, CF-200182, and CF-210766, that produce 2-(-hydroxy-5-methoxyphenoxy) acrylic acid, integrastin B and palmarumycin C15 dereplicated among other unknown natural products in the axenic controls. Among the group of extracts with broad spectrum antifungal plant pathogens and with activity only when obtained from the *B. cinerea* inhibited mycelium, the strains CF-116869, CF-096730, CF-195204 presented australifungin, botryendial phytotoxin, and tentatively cissetin.

Strains that produced compounds with specific activity to *M. grisea*, but that did not show induced SMs when co-cultured with *B. cinerea*, constituted the largest group of the positive microbial interactions. Extracts from these 25 confronted strains (data not shown) presented, when dereplicated by LCMS 3-O-acetyl-botcineric acid, botryendial phytotoxin, equisetin, enniantin I and G, 11,12-hydroxy-eudesm-4-en-3-one, cordyol C, illudin C3, illudinic acid, integrastin B, 7-chrolo-6-methoxy-mellein, *cis*-4-hydroxy-6-deoxy-scytalone or xylarine, and tentatively, botrystic acid B, botrydial, citreoviridin licicolinic acid A, 4-hydroxy-5-methyl-mellein.

### Metabolites Induced When Co-culturing with *B. cinerea*

The most significant group of results are represented by seven strains in which the co-culture induced the production of antifungal activities against human and plant fungal pathogens and an important number of new SMs were only detected in the extracts form the co-cultures. The most clearly effect was observed in strain CF-118005 producing a broad spectrum antifungal activity in co-culture against *A. fumigatus* and the three plant pathogens. The LC/MRMS profiles show the production of as many as 21 induced metabolites, including a molecule tentatively identified as SNF 4794-7. Similar observations were obtained for other strains such as in the strain CF-183212 with broad spectrum antifungal activity against *Candida albicans* and the three plant pathogens, presented induced metabolites in co-culture; Cordyol C and a component with formula C_15_H_16_O_4_ related to the component C_15_H_16_O_5_ found in axenic cultures was induced in the strain CF-187233, Other broad spectrum activity extracts against both *C. albicans* an *A. fumigatus* and the three human pathogens were obtained from the strain CF-189741 where we identified mycorrhizin A and a chlorinated C_14_H_15_ClO_4_ metabolite among 17 different induced components. Similarly, in the strains CF-246868, CF-090071, and CF-268787 multiple secondary metabolites were induced including some known compounds such as palmarumycins C7 and C15 and other preussomerin UV-like spectrum components, or penicillic acid. Further follow-up will be required to purify the individual components in these extracts and evaluate the antifungal spectrum of activity of these metabolites.

The analysis of the antifungal extracts that presented antifungal activity against all or individual plant pathogens has also shown the high number of new induced metabolites that be detected as result of the interaction. This study permitted to identify the production of known molecules such as chaetoxanthone C (CF-108442), palmarumycin C13 (CF-176883), secalonic acid C (CF-090258), components with no match in the Chapman & Hall Dictionary of Natural Products (DNPv25.1) such as the chlorated metabolite C_20_H_19_ClO_6_ (CF-108442) or C_11_H_15_ClO_5_ (CF-153629) not described previously for known fungal natural products, and of a large number of components ranging from 8 to 14 induced metabolites (CF-185636, CF-262323, CF-190679, CF-153629) that will require further characterization. The large number of specific activities specific to *M. grisea* (CF-090072, CF-090127, CF-110925, CF-118101, CF-195236, CF-209253, CF-213368, CF-284867, and CF-285353) and their lack of cytotoxicity that contrasts with the few specific activities obtained against *C. acutatum* or *F. proliferatum* suggests a higher sensitivity of the *M. grisea* assay to the presence of antifungals.

Given the high number of induced metabolites observed in the co-culturing study we focused the analysis on the most significant metabolite inductions. These are represented by 8 examples in which the induction of different SMs in extracts with antifungal against human and plant pathogens were detected in the inhibition zone (**Figure 4**).

In the case of the fungus *Lachunm* sp. CF-189741, the co-culture extract that presented a broad spectrum of activity against both *C. albicans* an *A. fumigatus* and the three plant pathogens showed among other 19 induced components a chlorinated metabolite (C_14_H_15_ClO_4_) and a significant increase in the production of the mycotoxin mycorrhizin A, when compared to the axenic culture. Mycorrhizin A was previously described from the ectendomycorrhizal fungus *Monotropa hypopitys* (Trofast and Wickberg, 1977) and later reported as the responsible for the antimicrobial activity of extracts from various fungal endophytes (Pimentel et al., 2010; Hussain et al., 2014) (**Figure 5A**).

Axenic cultures of the strain *A. nidulans* CF-090127 (**Figure 5B**) produced violaceol, whereas in co-culturing we observed the induction of diorcinol. Both SMs were previously isolated from a marine-derived isolate of *A. versicolor* and were shown to have antibiotic activities (Fremlin et al., 2009). Recent genome analysis of the *A. nidulans* has evidenced that the polyketide synthase *orsA*, a member of the F9775 secondary metabolite gene cluster, is required for the biosynthesis of F9775, orsellinic acid and violaceol (Nielsen et al., 2011), and deletions of other putative orselinic acid/F9775 cluster resulted in accumulation of the bioactive compounds gerfelin and diorcinol (Sanchez et al., 2010). Our data suggest that in this case the putative expression of this pathway may be repressed in the co-culturing, resulting in the formation of diorcinol and suppression of violaceol.

The strain *Penicillium* sp. CF-284867 was shown to produce griseofulvin and dechlorogriseofulvin when growth axenically, though in co-culturing the production of these two molecules was significantly increased, together with the induction of the mycotoxin cyclopiazonic acid (CPA) (**Figure 5C**). CPA has been described as the main toxic principle of a strain of *Penicillium cyclopium* (Holzapfel, 1968) but is also known to be produced by several species of *Aspergillus* and *Penicillium* (Frisvad et al., 2006). Griseofulvin was first isolated from *Penicillium griseofulvum* (Oxford et al., 1939) and later from other species of *Penicillium*, and is known to inhibit the mycelial growth of fungi but has no effect on yeasts. This fungistatic drug is used in animals and humans, in treatment of fungal infections of the skin and nail (Borgers, 1980). The co-culture induced natural product CPA is a known iron chelator and has been shown to be a specific inhibitor of Ca^2+^ ATPase of the sarcoplasmic reticulum (Seidler et al., 1989). The benefit of CPA to the producing fungi is not clear and there is no literature supporting specific antimicrobial activity.

In co-culture the endophyte *Stagonospora* sp. CF-090258 induced the production of secalonic acid C that showed activity against *C. acutatum* (**Figure 5D**). Secalonic acids are members of the ergochrome group of SMs, and can be isolated from the plant pathogenic fungus *Claviceps purpurea* as well as from other fungi, including *Aspergillus* spp. and *Penicillium* spp.

Among the molecules that were only produced in the zone of inhibition of the co-culture, we identified chaetoxanthone C as produced by the strain of *Verticillium* sp. CF-108442. This compound was only detected in the extract from the inhibition zone of the co-culture and the extract produced growth inhibition in all three plant pathogens. Xanthones are well known for their pharmacological activities (Hostettmann and Hostettmann, 1989) such as antibacterials and anti-fungals (Braz-Filho, 1999; Beerhues et al., 2000). Chaetoxanthone C has been previously described from the marine-derived fungus *Chaetomium* sp., with specific antimalarial activity and low cytotoxicity (Pontius et al., 2008), but this study is the first report of the production of this compound by a *Verticillium* sp. from a terrestrial origin (**Figure 5E**). Furthermore, our results are in line with a similar study of a co-culture broth of two marine fungi, and the induction of a new xanthone with antifungal activity (Li et al., 2011). Xanthones are known to be elicited in plants, where they play a dual function during biotic stress: as antioxidants to protect the cells from oxidative damage and as phytoalexins to impair the pathogen growth (Franklin et al., 2009). A similar role could be proposed for these compounds in fungal interactions.

The co-culture of the unidentified endophyte CF-195236 that showed a specific activity against *M. grisea* induced the production hydroxymellein, a metabolite not detected when the strain was grown axenically (**Figure 5F**). Hydroxymellein is a mellein derivative reported recently in antimicrobial extracts from fungal endophytes (Oliveira et al., 2009; Santiago et al., 2014) and our results suggest that the production of this compound can be the result of a crosstalk resulting from the induction of potentially silent biosynthetic gene clusters.

Another strain producing a specific activity against *M. grisea* is the *Phoma* sp. CF-090072. The strain is a prolific producer of new multi-chlorinated natural products (chemical formula with no match in DNP), most unusual in fungi. In axenic cultures, we only detected a low yield of the compounds C_15_H_13_ClO_5_ and C_15_H_12_Cl_2_O, while in co-cultures with *B. cinerea* the production titer for these compounds was significantly increased, and we could detect the induction of two additional multi-chlorinated compounds (C_20_H_14_Cl_3_O_5_ and C_19_H_12_Cl_5_O_3_) (**Figure 5G**). A search in DNP for fungal-derived polychlorinated natural products shows that only 64 were found to have three or more chlorides, and only 11 possess five or more chlorides. This is another example of unusual secondary metabolites that can be elicited by co-culturing, and efforts to scale up and isolate these chlorinated compounds are under way.

The extract of the co-culture of strain *Skeletocutis amorpha* CF-190679 that showed activity against *F. proliferatum* presented multiple components (12), many with molecular formulas not described for known natural products with no match in the Chapman & Hall Dictionary of Natural Products (DNPv25.1). The strain produced among others a possible new compound C_23_H_36_O_9_ when grown axenically and in co-culture, as well as a potential new compound C_40_H_62_O_13_ only induced in co-culture (**Figure 5H**). *Skeletocutis* is a genus of more than 60 species belonging to the Polyporales (Kirk et al., 2008) and key players in the carbon cycle, and the white-rot members of the order are among the most efficient lignin decayers in the biosphere (Floudas et al., 2012; Riley et al., 2014). To date natural product derived from fungal species have been reported so far and efforts to scale up and isolate such novel compounds without no match in DNP, are under way.

In most of the extracts the LC-MS analysis did not detect any known molecules that could explain the inhibitory activity, especially in the case of the extracts with specific activity against one of three phytopathogens. Even in the case of the production of preussomerin B y F, equisetin y griseofulvin, all well known as broad spectrum antifungal agents, we did not observe any inhibition of the growth of *C. acutatum* and *F. proliferatum*, an observation that can be related to the low concentration of these molecules below the minimal inhibitory concentration (MIC) for these phytopathogens.

In summary, follow-up analysis including co-cultivation scale-up, chemical fractionation and purification of several compounds from the positive microbial interactions are currently being performed for the identification of SMs responsible for their biological activities. This will permit to characterize the novelty of the compounds and the activity profile against these pathogens.

## Conclusion

Co-culturing with *B. cinerea* has proven to be a successful way of inducing antifungals with activity against both human and plant pathogens. The strain interaction determines dramatic changes in the number and levels of SMs detected with significative changes in the metabolite profiles.

Although *B. cinerea* is a good candidate in co-culturing experiments to induce new activities in the population of confronted strains, the highest number of positive antagonism compounds were obtained against plant pathogens, and only in a reduced number of cases we could confirm the production of antifungal activities against human pathogens. These results suggest that the selection of human pathogens such as *A. fumigatus* or *C. albicans* could be a more suitable co-culture inducer to trigger the production of new antifungals against human pathogens.

Further follow-up studies that are in progress in our laboratory to characterize the most interesting activities and potentially novel compound will permit to confirm the antifungal spectrum of these components and identify the diversity of novel components that have been elicited upon microbial interaction.

## Authors Contributions

VG-M, JT, OG designed the experiments; RS, LR and JM performed the experiments; RS, VG-M, and JT collected and analyzed the data; and VG-M, JT, and OG wrote the manuscript.

## Conflict of Interest Statement

The authors declare that the research was conducted in the absence of any commercial or financial relationships that could be construed as a potential conflict of interest.

## Footnotes

1 http://www.nbrc.nite.go.jp/
