# Supplementary material for: Co-culturing of Fungal Strains Against Botrytis cinerea as a Model for the Induction of Chemical Diversity and Therapeutic Agents
Source: Front Microbiol. 2017 Apr 19;8:649. doi: 10.3389/fmicb.2017.00649 (PMC5396503; doi:10.3389/fmicb.2017.00649)
Supplement: Supplementary file 1 [file Table_1.DOCX]

Supplementary Material

Co-Culturing of Fungal Strains Against *Botrytis cinerea* as a Model for the Induction of Chemical Diversity and Therapeutic Agents

Rachel Serrano, Víctor González-Menéndez, Lorena Rodríguez, Jesús Martín, José R. Tormo and Olga Genilloud*

*** Correspondence:** Corresponding Author: [olga.genilloud@medinaandalucia.es](mailto:olga.genilloud@medinaandalucia.es)

### Supplementary Data. Table 1. Results of antimicrobial assays against fungal pathogens and evaluation of cytotoxicity on HepG2 cell line. Positive results indicate percentage of cell over-growth of the strains. Negative results indicate percentage of cell growth-inhibition compared to standards.

| **Compound ID** | **Description** | **LCMS Match**  **with known compounds** | ***C. acutatum*** | ***F. proliferatum*** | ***M. grisea*** | ***C. albicans*** | ***A. fumigatus*** | **Hep-G2** |
| --- | --- | --- | --- | --- | --- | --- | --- | --- |
|  |  |  | **Inhibition zone (mm)** | **Inhibition zone (mm)** | **Inhibition zone (mm)** | **Inhibition zone (mm)** | **Inhibition zone (mm)** | **% Inh (24h)** |
| **C?-292032-a01-MO001-EC01** | **Control** |  | **0** | **0** | **0** | **0** | **0** | **113.91** |
| **C?-295211-a02-MO002-EC01** | **Control** |  | **0** | **0** | **0** | **0** | **0** | **48.98** |
| **CF-086331-a06-MO001-EC01** | **Axenic** | **Gliotoxin** | **7.6** | **0** | **0** | **0** | **0** | **-96.92** |
| **CF-086331-a06-MO002-EC01** | **Axenic** | **Gliotoxin** | **5.6** | **0** | **0** | **0** | **0** | **-85.12** |
| **CBS 102414/CF-086331-a03** | **Inhibition zone** |  | **0** | **0** | **6.7** | **0** | **0** | **-17.50** |
| **CBS 102414/CF-086331-a03** | **Inhibited mycelium** |  | **0** | **0** | **0** | **0** | **0** | **1.18** |
| **CF-090071-a05-MO001-EC01** | **Axenic** |  | **0** | **0** | **0** | **0** | **0** | **26.49** |
| **CF-090071-a05-MO002-EC01** | **Axenic** |  | **0** | **0** | **0** | **0** | **0** | **30.02** |
| **CBS 102414/CF-090071-a03** | **Inhibition zone** |  | **0** | **6.6** | **5.5** | **0** | **0** | **78.28** |
| **CBS 102414/CF-090071-a03** | **Inhibited mycelium** |  | **0** | **6.6** | **0** | **0** | **0** | **101.28** |
| **CF-090072-a04-MO001-EC01** | **Axenic** |  | **0** | **0** | **0** | **0** | **0** | **22.96** |
| **CF-090072-a04-MO002-EC01** | **Axenic** |  | **0** | **0** | **0** | **0** | **0** | **34.36** |
| **CBS 102414/CF-090072-a03** | **Inhibition zone** |  | **0** | **0** | **6.4** | **0** | **0** | **58.52** |
| **CBS 102414/CF-090072-a03** | **Inhibited mycelium** |  | **0** | **0** | **0** | **0** | **0** | **75.90** |
| **CF-090127-a02-MO002-EC01** | **Axenic** | **Violaceol I/ II** | **0** | **0** | **0** | **0** | **0** | **2.27** |
| **CF-090127-a02-MO003-EC01** | **Axenic** | **Violaceol I/ II** | **0** | **0** | **0** | **0** | **0** | **11.86** |
| **CBS 102414/CF-090127-a03** | **Inhibition zone** |  | **0** | **0** | **6.1** | **0** | **0** | **29.92** |
| **CBS 102414/CF-090127-a03** | **Inhibited mycelium** |  | **0** | **0** | **0** | **0** | **0** | **74.07** |
| **CF-090258-a07-MO001-EC01** | **Axenic** |  | **0** | **0** | **0** | **0** | **0** | **31.23** |
| **CF-090258-a07-MO002-EC01** | **Axenic** |  | **0** | **0** | **0** | **0** | **0** | **17.10** |
| **CBS 102414/CF-090258-a03** | **Inhibition zone** |  | **0** | **0** | **0** | **0** | **0** | **4.61** |
| **CBS 102414/CF-090258-a03** | **Inhibited mycelium** |  | **4.9** | **0** | **0** | **0** | **0** | **-66.78** |
| **CF-090378-a06-MO001-EC01** | **Axenic** |  | **0** | **0** | **0** | **0** | **0** | **22.65** |
| **CF-090378-a06-MO002-EC01** | **Axenic** |  | **0** | **0** | **0** | **0** | **0** | **28.91** |
| **CBS 102414/CF-090378-a05** | **Inhibition zone** |  | **0** | **0** | **6.9** | **0** | **0** | **90.79** |
| **CBS 102414/CF-090378-a05** | **Inhibited mycelium** |  | **0** | **0** | **0** | **0** | **0** | **61.79** |
| **CF-091927-a05-MO001-EC01** | **Axenic** |  | **0** | **0** | **0** | **0** | **0** | **3.56** |
| **CF-091927-a05-MO002-EC01** | **Axenic** |  | **0** | **0** | **0** | **0** | **0** | **-7.51** |
| **CBS 102414/CF-091927-a03** | **Inhibition zone** |  | **0** | **0** | **0** | **0** | **0** | **-27.30** |
| **CBS 102414/CF-091927-a03** | **Inhibited mycelium** |  | **0** | **0** | **6.4** | **0** | **0** | **20.64** |
| **CF-092670-a04-MO001-EC01** | **Axenic** |  | **0** | **0** | **0** | **0** | **0** | **25.68** |
| **CF-092670-a04-MO002-EC01** | **Axenic** |  | **0** | **0** | **0** | **0** | **0** | **40.41** |
| **CBS 102414/CF-092670-a03** | **Inhibition zone** |  | **0** | **0** | **0** | **0** | **0** | **-90.79** |
| **CBS 102414/CF-092670-a03** | **Inhibited mycelium** |  | **0** | **0** | **6.7** | **0** | **0** | **-95.10** |
| **CF-095017-a04-MO001-EC01** | **Axenic** |  | **0** | **0** | **0** | **0** | **0** | **25.88** |
| **CF-095017-a04-MO002-EC01** | **Axenic** |  | **0** | **0** | **0** | **0** | **0** | **19.42** |
| **CBS 102414/CF-095017-a03** | **Inhibition zone** |  | **0** | **0** | **0** | **0** | **0** | **66.75** |
| **CBS 102414/CF-095017-a03** | **Inhibited mycelium** |  | **0** | **0** | **7.2** | **0** | **0** | **-95.36** |
| **CF-096730-a08-MO001-EC01** | **Axenic** |  | **0** | **0** | **0** | **0** | **0** | **0.05** |
| **CF-096730-a08-MO002-EC01** | **Axenic** |  | **0** | **0** | **0** | **0** | **0** | **19.53** |
| **CBS 102414/CF-096730-a06** | **Inhibition zone** |  | **0** | **0** | **0** | **0** | **0** | **9.01** |
| **CBS 102414/CF-096730-a06** | **Inhibited mycelium** |  | **5.6** | **0** | **6.3** | **0** | **0** | **-23.07** |
| **CF-108442-a04-MO001-EC01** | **Axenic** |  | **0** | **0** | **0** | **0** | **0** | **7.11** |
| **CF-108442-a04-MO002-EC01** | **Axenic** |  | **0** | **0** | **0** | **0** | **0** | **-5.70** |
| **CBS 102414/CF-108442-a03** | **Inhibition zone** |  | **6.2** | **6.9** | **6.8** | **0** | **0** | **-40.10** |
| **CBS 102414/CF-108442-a03** | **Inhibited mycelium** |  | **0** | **0** | **5.9** | **0** | **0** | **-42.19** |
| **CF-109100-a04-MO001-EC01** | **Axenic** |  | **0** | **0** | **0** | **0** | **0** | **15.78** |
| **CF-109100-a04-MO002-EC01** | **Axenic** |  | **0** | **0** | **0** | **0** | **0** | **23.79** |
| **CBS 102414/CF-109100-a03** | **Inhibition zone** |  | **0** | **0** | **6.3** | **0** | **0** | **-23.91** |
| **CBS 102414/CF-109100-a03** | **Inhibited mycelium** |  | **0** | **0** | **0** | **0** | **0** | **-14.63** |
| **CF-109684-a10-MO001-EC01** | **Axenic** | **Gliotoxin** | **6.5** | **6.3** | **6.4** | **0** | **0** | **-99.95** |
| **CF-109684-a10-MO002-EC01** | **Axenic** | **Gliotoxin** | **5.5** | **0** | **0** | **0** | **0** | **-89.96** |
| **CBS 102414/CF-109684-a07** | **Inhibition zone** |  | **0** | **0** | **6.5** | **0** | **0** | **-47.55** |
| **CBS 102414/CF-109684-a07** | **Inhibited mycelium** |  | **0** | **0** | **0** | **0** | **0** | **-36.19** |
| **CF-110925-a04-MO001-EC01** | **Axenic** |  | **0** | **0** | **0** | **0** | **0** | **23.97** |
| **CF-110925-a04-MO002-EC01** | **Axenic** |  | **0** | **0** | **0** | **0** | **0** | **19.53** |
| **CBS 102414/CF-110925-a03** | **Inhibition zone** |  | **0** | **0** | **6.3** | **0** | **0** | **44.94** |
| **CBS 102414/CF-110925-a03** | **Inhibited mycelium** |  | **0** | **0** | **0** | **0** | **0** | **-34.49** |
| **CF-111323-a04-MO001-EC01** | **Axenic** | **Cyclosporin A** | **0** | **0** | **0** | **0** | **0** | **17.81** |
| **CF-111323-a04-MO002-EC01** | **Axenic** |  | **0** | **0** | **0** | **0** | **0** | **13.37** |
| **CBS 102414/CF-111323-a03** | **Inhibition zone** |  | **0** | **5.7** | **6.7** | **0** | **0** | **2.77** |
| **CBS 102414/CF-111323-a03** | **Inhibited mycelium** |  | **5.4** | **7.9** | **7.4** | **0** | **0** | **-94.75** |
| **CF-114839-a05-MO001-EC01** | **Axenic** |  | **5.9** | **0** | **5** | **0** | **0** | **-92.08** |
| **CF-114839-a05-MO002-EC01** | **Axenic** |  | **0** | **0** | **6.5** | **0** | **0** | **-78.46** |
| **CBS 102414/CF-114839-a03** | **Inhibition zone** |  | **0** | **0** | **5.5** | **0** | **0** | **-20.64** |
| **CBS 102414/CF-114839-a03** | **Inhibited mycelium** |  | **0** | **0** | **0** | **0** | **0** | **-15.68** |
| **CF-116114-a05-MO001-EC01** | **Axenic** |  | **0** | **0** | **0** | **0** | **0** | **12.97** |
| **CF-116114-a05-MO002-EC01** | **Axenic** |  | **0** | **0** | **0** | **0** | **0** | **30.93** |
| **CBS 102414/CF-116114-a04** | **Inhibition zone** |  | **0** | **0** | **0** | **0** | **0** | **29.17** |
| **CBS 102414/CF-116114-a04** | **Inhibited mycelium** |  | **0** | **0** | **0** | **0** | **0** | **56.56** |
| **CF-116317-a04-MO001-EC01** | **Axenic** |  | **6.5** | **6.3** | **6.1** | **0** | **0** | **-94.40** |
| **CF-116317-a04-MO002-EC01** | **Axenic** |  | **0** | **0** | **6.7** | **0** | **0** | **-94.30** |
| **CBS 102414/CF-116317-a03** | **Inhibition zone** |  | **0** | **0** | **0** | **0** | **0** | **-98.76** |
| **CBS 102414/CF-116317-a03** | **Inhibited mycelium** |  | **0** | **0** | **7.4** | **0** | **0** | **-99.93** |
| **CF-116676-a10-MO001-EC01** | **Axenic** | **Preussomerin B, Preussomerin A, Preussomerin L** | **7.1** | **7.5** | **6.7** | **7.7** | **10.2** | **-100.05** |
| **CF-116676-a10-MO002-EC01** | **Axenic** | **Preussomerin B, Preussomerin A, Preussomerin L** | **7.2** | **7.1** | **7.5** | **7.3** | **10.3** | **-99.95** |
| **CBS 102414/CF-116676-a07** | **Inhibition zone** | **Palmarumycin C15, Preussomerin B, F** | **6.1** | **7.5** | **9.8** | **0** | **10.7** | **-99.54** |
| **CBS 102414/CF-116676-a07** | **Inhibited mycelium** | **Palmarumycin C15, Preussomerin B, F** | **7** | **7** | **6.3** | **0** | **7.9** | **-99.67** |
| **CF-116728-a04-MO001-EC01** | **Axenic** |  | **0** | **0** | **0** | **0** | **0** | **-100.05** |
| **CF-116728-a04-MO002-EC01** | **Axenic** |  | **0** | **0** | **0** | **0** | **0** | **-90.16** |
| **CBS 102414/CF-116728-a03** | **Inhibition zone** |  | **5.6** | **6.2** | **8.7** | **0** | **0** | **-99.54** |
| **CBS 102414/CF-116728-a03** | **Inhibited mycelium** |  | **6.1** | **6.6** | **5.6** | **0** | **0** | **-93.14** |
| **CF-116869-a04-MO001-EC01** | **Axenic** |  | **0** | **0** | **0** | **0** | **0** | **40.82** |
| **CF-116869-a04-MO002-EC01** | **Axenic** |  | **0** | **0** | **0** | **0** | **0** | **37.69** |
| **CBS 102414/CF-116869-a03** | **Inhibition zone** |  | **0** | **0** | **0** | **0** | **0** | **32.29** |
| **CBS 102414/CF-116869-a03** | **Inhibited mycelium** |  | **6.4** | **6.1** | **6.3** | **0** | **0** | **-29.74** |
| **CF-118005-a04-MO001-EC01** | **Axenic** |  | **0** | **0** | **8.3** | **0** | **0** | **20.74** |
| **CF-118005-a04-MO002-EC01** | **Axenic** |  | **6.5** | **0** | **6.8** | **0** | **0** | **23.56** |
| **CBS 102414/CF-118005-a03** | **Inhibition zone** |  | **5.9** | **7.5** | **0** | **0** | **8.9** | **-97.98** |
| **CBS 102414/CF-118005-a03** | **Inhibited mycelium** |  | **7.3** | **7.9** | **6.1** | **0** | **7.5** | **-98.24** |
| **CF-118101-a05-MO001-EC01** | **Axenic** |  | **0** | **0** | **0** | **0** | **0** | **-17.00** |
| **CF-118101-a05-MO002-EC01** | **Axenic** |  | **0** | **0** | **0** | **0** | **0** | **-12.26** |
| **CBS 102414/CF-118101-a04** | **Inhibition zone** |  | **0** | **0** | **6.5** | **0** | **0** | **-22.60** |
| **CBS 102414/CF-118101-a04** | **Inhibited mycelium** |  | **0** | **0** | **0** | **0** | **0** | **-20.25** |
| **CF-153629-a03-MO001-EC01** | **Axenic** |  | **0** | **0** | **0** | **0** | **0** | **-7.11** |
| **CF-153629-a03-MO002-EC01** | **Axenic** |  | **0** | **0** | **0** | **0** | **0** | **-1.56** |
| **CBS 102414/CF-153629-a02** | **Inhibition zone** |  | **6.3** | **0** | **0** | **0** | **0** | **0.92** |
| **CBS 102414/CF-153629-a02** | **Inhibited mycelium** |  | **0** | **0** | **0** | **0** | **0** | **-49.04** |
| **CF-160675-a04-MO001-EC01** | **Axenic** |  | **0** | **0** | **0** | **0** | **0** | **9.13** |
| **CF-160675-a04-MO002-EC01** | **Axenic** |  | **0** | **0** | **0** | **0** | **0** | **29.52** |
| **CBS 102414/CF-160675-a03** | **Inhibition zone** |  | **0** | **0** | **0** | **0** | **0** | **82.40** |
| **CBS 102414/CF-160675-a03** | **Inhibited mycelium** |  | **0** | **0** | **10** | **0** | **0** | **17.67** |
| **CF-164326-a07-MO001-EC01** | **Axenic** | **Preussomerin B, Preussomerin L** | **5.9** | **7.1** | **6** | **0** | **9.2** | **-95.41** |
| **CF-164326-a07-MO002-EC01** | **Axenic** | **Preussomerin B, Preussomerin L** | **0** | **0** | **0** | **0** | **0** | **-71.49** |
| **CBS 102414/CF-164326-a04** | **Inhibition zone** | **Preussomerin B** | **6** | **0** | **6.1** | **0** | **0** | **-84.39** |
| **CBS 102414/CF-164326-a04** | **Inhibited mycelium** | **Preussomerin B** | **0** | **0** | **0** | **0** | **0** | **-84.00** |
| **CF-175615-a05-MO001-EC01** | **Axenic** | **Preussomerin L** | **7.3** | **7.1** | **6.4** | **0** | **9.7** | **-98.44** |
| **CF-175615-a05-MO002-EC01** | **Axenic** | **Preussomerin A, Preussomerin L** | **6.7** | **7** | **6.8** | **0** | **8.8** | **-97.83** |
| **CBS 102414/CF-175615-a03** | **Inhibition zone** | **Preussomerin B, F** | **7.3** | **7** | **7** | **0** | **9** | **-98.89** |
| **CBS 102414/CF-175615-a03** | **Inhibited mycelium** | **Preussomerin B, F** | **6.6** | **7.8** | **7.2** | **0** | **8** | **-98.89** |
| **CF-175637-a05-MO001-EC01** | **Axenic** | **Preussomerin L** | **7.4** | **7.1** | **7.2** | **9.8** | **10.8** | **-99.85** |
| **CF-175637-a05-MO002-EC01** | **Axenic** | **Preussomerin L** | **6.5** | **7.7** | **6.5** | **0** | **9** | **-93.59** |
| **CBS 102414/CF-175637-a03** | **Inhibition zone** | **Palmarumycin C15, Preussomerin B, F** | **7.2** | **8.3** | **6.5** | **0** | **7.7** | **-49.61** |
| **CBS 102414/CF-175637-a03** | **Inhibited mycelium** | **Palmarumycin C15, Preussomerin B, F** | **7.3** | **7.5** | **6.1** | **0** | **8.2** | **-98.01** |
| **CF-176883-a05-MO001-EC01** | **Axenic** |  | **0** | **0** | **0** | **0** | **0** | **-4.59** |
| **CF-176883-a05-MO002-EC01** | **Axenic** |  | **0** | **0** | **0** | **0** | **0** | **27.70** |
| **CBS 102414/CF-176883-a03** | **Inhibition zone** |  | **7** | **6.4** | **5.5** | **0** | **0** | **-80.41** |
| **CBS 102414/CF-176883-a03** | **Inhibited mycelium** |  | **7** | **6.9** | **7** | **0** | **0** | **-96.03** |
| **CF-177133-a05-MO001-EC01** | **Axenic** |  | **0** | **0** | **6.6** | **0** | **0** | **17.20** |
| **CF-177133-a05-MO002-EC01** | **Axenic** |  | **0** | **0** | **0** | **0** | **0** | **6.41** |
| **CBS 102414/CF-177133-a03** | **Inhibition zone** |  | **6.1** | **6.3** | **6.3** | **0** | **0** | **-96.03** |
| **CBS 102414/CF-177133-a03** | **Inhibited mycelium** |  | **6.7** | **7.3** | **6** | **0** | **0** | **-97.30** |
| **CF-179292-a05-MO001-EC01** | **Axenic** |  | **6.2** | **0** | **0** | **0** | **0** | **-0.66** |
| **CF-179292-a05-MO002-EC01** | **Axenic** |  | **0** | **0** | **0** | **0** | **0** | **9.43** |
| **CBS 102414/CF-179292-a02** | **Inhibition zone** |  | **0** | **0** | **6** | **0** | **0** | **24.69** |
| **CBS 102414/CF-179292-a02** | **Inhibited mycelium** |  | **0** | **0** | **0** | **0** | **0** | **-14.11** |
| **CF-182855-a07-MO001-EC01** | **Axenic** |  | **0** | **0** | **0** | **0** | **0** | **11.65** |
| **CF-182855-a07-MO002-EC01** | **Axenic** |  | **0** | **0** | **0** | **0** | **0** | **24.57** |
| **CBS 102414/CF-182855-a04** | **Inhibition zone** | **Equisetin** | **0** | **0** | **6.1** | **0** | **0** | **12.70** |
| **CBS 102414/CF-182855-a04** | **Inhibited mycelium** | **Equisetin** | **0** | **0** | **0** | **0** | **0** | **-9.01** |
| **CF-183032-a05-MO001-EC01** | **Axenic** | **Preussomerin A, Preussomerin L** | **6.4** | **6.7** | **6.3** | **0** | **7.8** | **-94.40** |
| **CF-183032-a05-MO002-EC01** | **Axenic** | **Preussomerin A, Preussomerin L** | **0** | **6.2** | **6.7** | **0** | **0** | **-70.18** |
| **CBS 102414/CF-183032-a03** | **Inhibition zone** | **Preussomerin B, F** | **7.5** | **6.7** | **0** | **0** | **6** | **-76.68** |
| **CBS 102414/CF-183032-a03** | **Inhibited mycelium** | **Preussomerin F** | **6.6** | **6.9** | **6.8** | **0** | **6.9** | **-82.82** |
| **CF-183212-a07-MO001-EC01** | **Axenic** |  | **0** | **0** | **0** | **0** | **0** | **-20.03** |
| **CF-183212-a07-MO002-EC01** | **Axenic** |  | **0** | **0** | **0** | **0** | **0** | **15.39** |
| **CBS 102414/CF-183212-a04** | **Inhibition zone** |  | **6.3** | **6.6** | **7.4** | **6.2** | **0** | **-97.71** |
| **CBS 102414/CF-183212-a04** | **Inhibited mycelium** |  | **6** | **6.7** | **7.1** | **0** | **0** | **-95.23** |
| **CF-185390-a05-MO001-EC01** | **Axenic** |  | **0** | **0** | **0** | **0** | **0** | **-9.13** |
| **CF-185390-a05-MO002-EC01** | **Axenic** |  | **0** | **0** | **0** | **0** | **0** | **-9.94** |
| **CBS 102414/CF-185390-a03** | **Inhibition zone** | **Verscenoside C** | **0** | **0** | **0** | **0** | **0** | **47.76** |
| **CBS 102414/CF-185390-a03** | **Inhibited mycelium** | **Verscenoside C** | **0** | **0** | **0** | **0** | **0** | **10.01** |
| **CF-185391-a05-MO001-EC01** | **Axenic** |  | **0** | **0** | **0** | **0** | **0** | **-7.72** |
| **CF-185391-a05-MO002-EC01** | **Axenic** |  | **0** | **0** | **0** | **0** | **0** | **12.97** |
| **CBS 102414/CF-185391-a03** | **Inhibition zone** |  | **6.1** | **0** | **6.6** | **0** | **0** | **-92.62** |
| **CBS 102414/CF-185391-a03** | **Inhibited mycelium** |  | **5** | **6.9** | **7.1** | **0** | **0** | **-91.84** |
| **CF-185405-a06-MO001-EC01** | **Axenic** | **Preussomerin L** | **6.5** | **6.9** | **5.9** | **0** | **11.3** | **-99.04** |
| **CF-185405-a06-MO002-EC01** | **Axenic** | **Preussomerin L** | **5.8** | **6.1** | **6.9** | **0** | **8.1** | **-94.80** |
| **CBS 102414/CF-185405-a04** | **Inhibition zone** | **Preussomerin F** | **7.1** | **7.3** | **7.6** | **0** | **10.8** | **-99.28** |
| **CBS 102414/CF-185405-a04** | **Inhibited mycelium** | **Preussomerin B, F** | **6.5** | **7.4** | **7.8** | **0** | **10.1** | **-99.93** |
| **CF-185415-a07-MO001-EC01** | **Axenic** | **Preussomerin B, Preussomerin A, Preussomerin L** | **7.1** | **8.4** | **8.2** | **8.8** | **14.9** | **-99.75** |
| **CF-185415-a07-MO002-EC01** | **Axenic** | **Preussomerin B, Preussomerin A, Preussomerin L** | **5.1** | **7.3** | **6.7** | **0** | **10.3** | **-98.23** |
| **CBS 102414/CF-185415-a05** | **Inhibition zone** | **Preussomerin B, F** | **6.5** | **7.4** | **6.2** | **0** | **8.8** | **-98.44** |
| **CBS 102414/CF-185415-a05** | **Inhibited mycelium** | **Preussomerin B, F** | **6.7** | **7.5** | **6.2** | **0** | **9.6** | **-95.17** |
| **CF-185600-a05-MO001-EC01** | **Axenic** | **Preussomerin L** | **0** | **6.5** | **6** | **0** | **9.2** | **-93.09** |
| **CF-185600-a05-MO002-EC01** | **Axenic** | **Preussomerin L** | **0** | **7** | **6.9** | **0** | **9.2** | **-90.97** |
| **CBS 102414/CF-185600-a03** | **Inhibition zone** |  | **6.5** | **5.7** | **6.1** | **0** | **0** | **-67.02** |
| **CBS 102414/CF-185600-a03** | **Inhibited mycelium** | **Preussomerin F** | **6.7** | **6.9** | **6.8** | **0** | **8.2** | **-99.41** |
| **CF-185603-a08-MO001-EC01** | **Axenic** | **Preussomerin L** | **6.2** | **7.1** | **6.4** | **0** | **8.4** | **-96.42** |
| **CF-185603-a08-MO002-EC01** | **Axenic** | **Preussomerin L** | **0** | **6.8** | **6.8** | **0** | **5.9** | **-86.73** |
| **CBS 102414/CF-185603-a03** | **Inhibition zone** | **Preussomerin F** | **6.8** | **6.8** | **7.1** | **0** | **7.1** | **-79.69** |
| **CBS 102414/CF-185603-a03** | **Inhibited mycelium** | **Preussomerin F** | **7.1** | **7.4** | **0** | **0** | **8.4** | **-94.06** |
| **CF-185636-a05-MO001-EC01** | **Axenic** | **C_20_H_14_O_4_+ C_20_H_16_O_5_** | **6.6** | **6.6** | **6** | **0** | **0** | **-99.85** |
| **CF-185636-a05-MO002-EC01** | **Axenic** | **C_20_H_14_O_4_+ C_20_H_16_O_5_** | **0** | **6.1** | **6.2** | **0** | **0** | **-99.95** |
| **CBS 102414/CF-185636-a03** | **Inhibition zone** | **C_20_H_14_O_4_+ C_20_H_16_O_5_** | **6.8** | **6.7** | **6.6** | **0** | **0** | **-98.86** |
| **CBS 102414/CF-185636-a03** | **Inhibited mycelium** | **C_20_H_14_O_4_+ C_20_H_16_O_5_** | **6.5** | **0** | **0** | **0** | **0** | **-63.95** |
| **CF-185650-a05-MO001-EC01** | **Axenic** | **Preussomerin L** | **5.9** | **6.3** | **7.2** | **0** | **7.8** | **-82.09** |
| **CF-185650-a05-MO002-EC01** | **Axenic** |  | **0** | **0** | **0** | **0** | **0** | **-41.32** |
| **CBS 102414/CF-185650-a03** | **Inhibition zone** | **Palmarumycin C15, Preussomerin F** | **7.3** | **6.6** | **0** | **0** | **11** | **-95.03** |
| **CBS 102414/CF-185650-a03** | **Inhibited mycelium** | **Preussomerin F** | **6.9** | **8** | **6.6** | **0** | **8.4** | **-93.47** |
| **CF-185653-a06-MO001-EC01** | **Axenic** | **Cercosporamide** | **0** | **0** | **0** | **0** | **0** | **-10.71** |
| **CF-185653-a06-MO002-EC01** | **Axenic** | **Cercosporamide** | **0** | **0** | **0** | **0** | **0** | **17.33** |
| **CBS 102414/CF-185653-a03** | **Inhibition zone** |  | **6.4** | **0** | **5.7** | **0** | **0** | **-91.20** |
| **CBS 102414/CF-185653-a03** | **Inhibited mycelium** |  | **0** | **0** | **0** | **0** | **0** | **-54.15** |
| **CF-185670-a05-MO001-EC01** | **Axenic** |  | **0** | **0** | **0** | **0** | **0** | **4.29** |
| **CF-185670-a05-MO002-EC01** | **Axenic** |  | **0** | **0** | **0** | **0** | **0** | **13.50** |
| **CBS 102414/CF-185670-a03** | **Inhibition zone** |  | **0** | **0** | **7.2** | **0** | **0** | **4.96** |
| **CBS 102414/CF-185670-a03** | **Inhibited mycelium** |  | **0** | **0** | **0** | **0** | **0** | **27.30** |
| **CF-187233-a05-MO001-EC01** | **Axenic** |  | **0** | **0** | **0** | **0** | **0** | **-15.57** |
| **CF-187233-a05-MO002-EC01** | **Axenic** |  | **0** | **0** | **0** | **0** | **0** | **12.88** |
| **CBS 102414/CF-187233-a03** | **Inhibition zone** |  | **0** | **0** | **0** | **0** | **0** | **52.87** |
| **CBS 102414/CF-187233-a03** | **Inhibited mycelium** |  | **6.4** | **6.2** | **5.3** | **5.1** | **0** | **12.56** |
| **CF-187272-a06-MO001-EC01** | **Axenic** | **Palmarumycin C15, Preussomerin A, Preussomerin F, Preussomerin L** | **5.5** | **6.6** | **7.1** | **7.2** | **9.2** | **-99.69** |
| **CF-187272-a06-MO002-EC01** | **Axenic** | **Preussomerin L** | **6.2** | **6.3** | **7** | **0** | **8.4** | **-99.17** |
| **CBS 102414/CF-187272-a04** | **Inhibition zone** | **Palmarumycin C15, Preussomerin A** | **7** | **7.2** | **6.4** | **0** | **9** | **-97.59** |
| **CBS 102414/CF-187272-a04** | **Inhibited mycelium** | **Palmarumycin C15, Preussomerin F** | **7.4** | **7.4** | **0** | **0** | **7.3** | **-94.32** |
| **CF-187525-a05-MO001-EC01** | **Axenic** |  | **0** | **0** | **0** | **0** | **0** | **13.09** |
| **CF-187525-a05-MO002-EC01** | **Axenic** |  | **0** | **0** | **0** | **0** | **0** | **35.64** |
| **CBS 102414/CF-187525-a03** | **Inhibition zone** |  | **0** | **0** | **0** | **0** | **0** | **19.26** |
| **CBS 102414/CF-187525-a03** | **Inhibited mycelium** |  | **0** | **0** | **6** | **0** | **0** | **-17.04** |
| **CF-187689-a05-MO001-EC01** | **Axenic** |  | **0** | **0** | **0** | **0** | **0** | **-99.59** |
| **CF-187689-a05-MO002-EC01** | **Axenic** |  | **0** | **0** | **0** | **0** | **0** | **-97.72** |
| **CBS 102414/CF-187689-a03** | **Inhibition zone** |  | **0** | **0** | **6.1** | **0** | **0** | **19.46** |
| **CBS 102414/CF-187689-a03** | **Inhibited mycelium** |  | **0** | **0** | **0** | **0** | **0** | **-21.95** |
| **CF-187925-a05-MO001-EC01** | **Axenic** | **Palmarumycin C15, Preussomerin B, Preussomerin A, Preussomerin F, Preussomerin L** | **6.2** | **7.3** | **6.6** | **0** | **6.8** | **-99.59** |
| **CF-187925-a05-MO002-EC01** | **Axenic** | **Palmarumycin C15, Preussomerin B, Preussomerin A, Preussomerin F** | **0** | **7** | **0** | **0** | **0** | **-97.21** |
| **CBS 102414/CF-187925-a03** | **Inhibition zone** |  | **0** | **0** | **0** | **0** | **0** | **3.79** |
| **CBS 102414/CF-187925-a03** | **Inhibited mycelium** | **Preussomerin B, F** | **0** | **0** | **7.4** | **0** | **0** | **-12.67** |
| **CF-189741-a06-MO001-EC01** | **Axenic** | **Mycorrhizin A** | **5.2** | **5.9** | **5.7** | **0** | **7** | **-99.48** |
| **CF-189741-a06-MO002-EC01** | **Axenic** | **Mycorrhizin A** | **0** | **0** | **0** | **0** | **0** | **-19.09** |
| **CBS 102414/CF-189741-a03** | **Inhibition zone** | **Mycorrhizin A** | **9.5** | **8.5** | **9.3** | **7.9** | **9.4** | **-99.41** |
| **CBS 102414/CF-189741-a03** | **Inhibited mycelium** | **Mycorrhizin A** | **8.5** | **7.9** | **8.3** | **7.3** | **8.4** | **-99.26** |
| **CF-190679-a05-MO001-EC01** | **Axenic** |  | **0** | **0** | **0** | **0** | **0** | **-5.74** |
| **CF-190679-a05-MO002-EC01** | **Axenic** |  | **0** | **0** | **0** | **0** | **0** | **14.64** |
| **CBS 102414/CF-190679-a03** | **Inhibition zone** |  | **0** | **6.7** | **0** | **0** | **0** | **-99.67** |
| **CBS 102414/CF-190679-a03** | **Inhibited mycelium** |  | **0** | **0** | **7.9** | **0** | **0** | **37.62** |
| **CF-191074-a06-MO001-EC01** | **Axenic** | **Preussomerin B, Preussomerin F, Preussomerin L** | **6.4** | **7.6** | **6.1** | **0** | **8.4** | **-100.00** |
| **CF-191074-a06-MO002-EC01** | **Axenic** | **Preussomerin B, Preussomerin A, Preussomerin L** | **6.9** | **6.2** | **6** | **0** | **7.7** | **-99.90** |
| **CBS 102414/CF-191074-a04** | **Inhibition zone** | **Preussomerin B, F** | **6.5** | **7.8** | **0** | **0** | **8.3** | **-96.41** |
| **CBS 102414/CF-191074-a04** | **Inhibited mycelium** | **Preussomerin B, F** | **6.4** | **7.2** | **6.5** | **0** | **6.1** | **-99.15** |
| **CF-191080-a04-MO001-EC01** | **Axenic** | **Preussomerin B, Preussomerin A, Preussomerin L** | **6.5** | **7.9** | **6.3** | **0** | **10.7** | **-99.69** |
| **CF-191080-a04-MO002-EC01** | **Axenic** | **Preussomerin B, Preussomerin A, Preussomerin L** | **6.6** | **7.6** | **6.7** | **0** | **8.3** | **-99.59** |
| **CBS 102414/CF-191080-a03** | **Inhibition zone** | **Preussomerin B, F** | **6.8** | **6.9** | **0** | **0** | **6.6** | **-98.76** |
| **CBS 102414/CF-191080-a03** | **Inhibited mycelium** | **Preussomerin B, F** | **0** | **0** | **6.7** | **0** | **0** | **-99.93** |
| **CF-192432-a06-MO001-EC01** | **Axenic** | **Preussomerin A, Preussomerin L** | **6.1** | **8.3** | **6.6** | **0** | **8.7** | **-93.17** |
| **CF-192432-a06-MO002-EC01** | **Axenic** | **Preussomerin B, Preussomerin L, Preussomerin A** | **6.5** | **6.2** | **7.1** | **0** | **9.5** | **-96.38** |
| **CBS 102414/CF-192432-a04** | **Inhibition zone** | **Palmarumicin C15** | **6.2** | **5.9** | **5.1** | **0** | **7.9** | **-99.26** |
| **CBS 102414/CF-192432-a04** | **Inhibited mycelium** | **Palmarumicin C15** | **6** | **6.5** | **5.7** | **0** | **8** | **-99.41** |
| **CF-192817-a07-MO001-EC01** | **Axenic** |  | **6.7** | **0** | **7.1** | **0** | **0** | **-55.61** |
| **CF-192817-a07-MO002-EC01** | **Axenic** |  | **0** | **0** | **0** | **0** | **0** | **-2.43** |
| **CBS 102414/CF-192817-a03** | **Inhibition zone** |  | **0** | **0** | **6.8** | **0** | **0** | **75.90** |
| **CBS 102414/CF-192817-a03** | **Inhibited mycelium** |  | **0** | **0** | **0** | **0** | **0** | **15.55** |
| **CF-192842-a05-MO001-EC01** | **Axenic** |  | **0** | **0** | **0** | **0** | **0** | **27.06** |
| **CF-192842-a05-MO002-EC01** | **Axenic** |  | **5.7** | **0** | **0** | **0** | **0** | **29.33** |
| **CBS 102414/CF-192842-a03** | **Inhibition zone** |  | **5.8** | **6.6** | **6.3** | **0** | **0** | **-95.23** |
| **CBS 102414/CF-192842-a03** | **Inhibited mycelium** |  | **0** | **0** | **0** | **0** | **0** | **-18.29** |
| **CF-195204-a05-MO001-EC01** | **Axenic** |  | **0** | **0** | **0** | **0** | **0** | **49.09** |
| **CF-195204-a05-MO002-EC01** | **Axenic** |  | **0** | **0** | **0** | **0** | **0** | **35.75** |
| **CBS 102414/CF-195204-a03** | **Inhibition zone** |  | **0** | **0** | **6.8** | **0** | **0** | **29.39** |
| **CBS 102414/CF-195204-a03** | **Inhibited mycelium** |  | **0** | **6.9** | **6.4** | **0** | **0** | **-67.15** |
| **CF-195236-a04-MO001-EC01** | **Axenic** | **Mycophenolic acid** | **5.8** | **5.9** | **5.4** | **0** | **0** | **-25.71** |
| **CF-195236-a04-MO002-EC01** | **Axenic** | **Mycophenolic acid** | **0** | **0** | **0** | **0** | **0** | **20.43** |
| **CBS 102414/CF-195236-a03** | **Inhibition zone** |  | **0** | **0** | **5** | **0** | **0** | **-26.67** |
| **CBS 102414/CF-195236-a03** | **Inhibited mycelium** |  | **0** | **0** | **0** | **0** | **0** | **19.26** |
| **CF-197986-a06-MO001-EC01** | **Axenic** | **Palmarumycin C15, Preussomerin L, Preussomerin B, Preussomerin A, Preussomerin F** | **6.7** | **7.5** | **5.8** | **8.4** | **9.7** | **-100.00** |
| **CF-197986-a06-MO002-EC01** | **Axenic** | **Palmarumycin C15, Preussomerin B, Preussomerin A, Preussomerin L** | **6.9** | **7.9** | **6.8** | **7.8** | **10.1** | **-99.90** |
| **CBS 102414/CF-197986-a05** | **Inhibition zone** | **Preussomerin B** | **6.1** | **7.4** | **6.9** | **0** | **9.9** | **-99.15** |
| **CBS 102414/CF-197986-a05** | **Inhibited mycelium** | **Preussomerin B** | **5.8** | **7.6** | **0** | **0** | **7.9** | **-99.54** |
| **CF-199866-a06-MO001-EC01** | **Axenic** | **Palmarumycin C15, Preussomerin A, Preussomerin L** | **5.9** | **6.3** | **6.4** | **0** | **8.9** | **-95.76** |
| **CF-199866-a06-MO002-EC01** | **Axenic** | **Palmarumycin C15, Preussomerin A, Preussomerin L** | **6.4** | **6.9** | **6.7** | **0** | **8** | **-95.55** |
| **CBS 102414/CF-199866-a04** | **Inhibition zone** | **Preussomerin F** | **6.3** | **6.8** | **0** | **0** | **8.6** | **-71.06** |
| **CBS 102414/CF-199866-a04** | **Inhibited mycelium** | **Palmarumycin C15, Preussomerin B, F** | **7.4** | **7.1** | **6.6** | **0** | **8.3** | **-97.32** |
| **CF-200182-a06-MO001-EC01** | **Axenic** |  | **0** | **0** | **0** | **0** | **0** | **11.74** |
| **CF-200182-a06-MO002-EC01** | **Axenic** |  | **0** | **0** | **0** | **0** | **0** | **28.71** |
| **CBS 102414/CF-200182-a03** | **Inhibition zone** |  | **0** | **7.4** | **5.3** | **0** | **0** | **-46.07** |
| **CBS 102414/CF-200182-a03** | **Inhibited mycelium** |  | **6.1** | **6** | **7** | **0** | **0** | **-99.56** |
| **CF-204001-a05-MO001-EC01** | **Axenic** |  | **0** | **0** | **0** | **0** | **0** | **5.85** |
| **CF-204001-a05-MO002-EC01** | **Axenic** |  | **0** | **0** | **0** | **0** | **0** | **5.23** |
| **CBS 102414/CF-204001-a03** | **Inhibition zone** |  | **0** | **0** | **0** | **0** | **0** | **60.09** |
| **CBS 102414/CF-204001-a03** | **Inhibited mycelium** |  | **0** | **0** | **8.2** | **0** | **0** | **36.84** |
| **CF-204100-a04-MO001-EC01** | **Axenic** |  | **0** | **0** | **0** | **0** | **0** | **3.16** |
| **CF-204100-a04-MO002-EC01** | **Axenic** |  | **4.9** | **0** | **0** | **0** | **0** | **12.47** |
| **CBS 102414/CF-204100-a03** | **Inhibition zone** |  | **0** | **0** | **0** | **0** | **0** | **74.07** |
| **CBS 102414/CF-204100-a03** | **Inhibited mycelium** |  | **0** | **7.4** | **6.8** | **0** | **0** | **-16.85** |
| **CF-204209-a04-MO001-EC01** | **Axenic** | **Trichothecin** | **7.2** | **7** | **6.8** | **0** | **7** | **-28.71** |
| **CF-204209-a04-MO002-EC01** | **Axenic** | **Trichothecin** | **6.2** | **7.8** | **7.2** | **0** | **0** | **-38.75** |
| **CBS 102414/CF-204209-a03** | **Inhibition zone** | **Tricothecin** | **7.7** | **6.1** | **9.6** | **7.6** | **8.1** | **4.96** |
| **CBS 102414/CF-204209-a03** | **Inhibited mycelium** | **Tricothecin** | **7.7** | **7.5** | **7** | **7.2** | **6.9** | **-5.49** |
| **CF-208279-a06-MO001-EC01** | **Axenic** |  | **0** | **0** | **0** | **0** | **0** | **11.54** |
| **CF-208279-a06-MO002-EC01** | **Axenic** |  | **0** | **0** | **0** | **0** | **0** | **22.09** |
| **CBS 102414/CF-208279-a03** | **Inhibition zone** |  | **0** | **0** | **6.5** | **0** | **0** | **-33.71** |
| **CBS 102414/CF-208279-a03** | **Inhibited mycelium** |  | **6.4** | **6.5** | **6.7** | **0** | **0** | **-99.43** |
| **CF-209171-a09-MO001-EC01** | **Axenic** |  | **0** | **0** | **0** | **0** | **0** | **2.33** |
| **CF-209171-a09-MO002-EC01** | **Axenic** |  | **0** | **0** | **0** | **0** | **0** | **13.09** |
| **CBS 102414/CF-209171-a07** | **Inhibition zone** |  | **0** | **0** | **6** | **0** | **0** | **-31.11** |
| **CBS 102414/CF-209171-a07** | **Inhibited mycelium** |  | **5** | **6.1** | **6** | **0** | **0** | **-84.00** |
| **CF-209253-a05-MO001-EC01** | **Axenic** |  | **0** | **0** | **0** | **0** | **0** | **-17.02** |
| **CF-209253-a05-MO002-EC01** | **Axenic** |  | **0** | **0** | **0** | **0** | **0** | **-5.95** |
| **CBS 102414/CF-209253-a03** | **Inhibition zone** |  | **0** | **0** | **6.3** | **0** | **0** | **82.81** |
| **CBS 102414/CF-209253-a03** | **Inhibited mycelium** |  | **0** | **0** | **0** | **0** | **0** | **20.59** |
| **CF-209628-a03-MO001-EC01** | **Axenic** |  | **0** | **0** | **0** | **0** | **0** | **25.30** |
| **CF-209628-a03-MO002-EC01** | **Axenic** |  | **0** | **0** | **0** | **0** | **0** | **16.92** |
| **CBS 102414/CF-209628-a02** | **Inhibition zone** |  | **0** | **0** | **0** | **0** | **0** | **17.96** |
| **CBS 102414/CF-209628-a02** | **Inhibited mycelium** |  | **0** | **0** | **0** | **0** | **0** | **17.81** |
| **CF-210766-a05-MO001-EC01** | **Axenic** |  | **0** | **0** | **0** | **0** | **0** | **12.05** |
| **CF-210766-a05-MO002-EC01** | **Axenic** |  | **0** | **0** | **0** | **0** | **0** | **17.12** |
| **CBS 102414/CF-210766-a03** | **Inhibition zone** | **Verscenoside C** | **0** | **6.5** | **5.4** | **0** | **0** | **-59.83** |
| **CBS 102414/CF-210766-a03** | **Inhibited mycelium** | **Verscenoside C** | **0** | **0** | **0** | **0** | **0** | **-16.25** |
| **CF-213368-a04-MO001-EC01** | **Axenic** |  | **0** | **0** | **0** | **0** | **0** | **-47.44** |
| **CF-213368-a04-MO002-EC01** | **Axenic** |  | **0** | **0** | **0** | **0** | **0** | **-38.44** |
| **CBS 102414/CF-213368-a03** | **Inhibition zone** |  | **0** | **0** | **6.1** | **0** | **0** | **-24.44** |
| **CBS 102414/CF-213368-a03** | **Inhibited mycelium** |  | **0** | **0** | **0** | **0** | **0** | **-9.04** |
| **CF-213376-a04-MO001-EC01** | **Axenic** |  | **0** | **0** | **0** | **0** | **0** | **-19.40** |
| **CF-213376-a04-MO002-EC01** | **Axenic** |  | **0** | **0** | **0** | **0** | **0** | **5.54** |
| **CBS 102414/CF-213376-a03** | **Inhibition zone** |  | **0** | **0** | **0** | **0** | **0** | **36.83** |
| **CBS 102414/CF-213376-a03** | **Inhibited mycelium** |  | **0** | **0** | **0** | **0** | **0** | **22.07** |
| **CF-214807-a04-MO001-EC01** | **Axenic** |  | **0** | **0** | **0** | **0** | **0** | **5.12** |
| **CF-214807-a04-MO002-EC01** | **Axenic** |  | **0** | **0** | **0** | **0** | **0** | **6.88** |
| **CBS 102414/CF-214807-a03** | **Inhibition zone** |  | **0** | **0** | **0** | **0** | **0** | **93.19** |
| **CBS 102414/CF-214807-a03** | **Inhibited mycelium** |  | **0** | **0** | **7.4** | **0** | **0** | **-10.37** |
| **CF-215288-a07-MO001-EC01** | **Axenic** |  | **4.7** | **0** | **0** | **0** | **0** | **10.50** |
| **CF-215288-a07-MO002-EC01** | **Axenic** |  | **0** | **0** | **0** | **0** | **0** | **-2.74** |
| **CBS 102414/CF-215288-a03** | **Inhibition zone** |  | **7.6** | **6.5** | **8.9** | **6.6** | **7.5** | **14.55** |
| **CBS 102414/CF-215288-a03** | **Inhibited mycelium** |  | **7** | **6.5** | **11.7** | **4.8** | **0** | **-17.96** |
| **CF-223912-a07-MO001-EC01** | **Axenic** | **Preussomerin B, Preussomerin L** | **5.5** | **7.9** | **6.8** | **7.7** | **12.8** | **-99.79** |
| **CF-223912-a07-MO002-EC01** | **Axenic** | **Preussomerin L** | **6.2** | **7.9** | **7.1** | **6.5** | **10.5** | **-98.65** |
| **CBS 102414/CF-223912-a06** | **Inhibition zone** | **Palmarumycin C15, Preussomerin F** | **8.1** | **7.9** | **11.2** | **9** | **17.8** | **-98.44** |
| **CBS 102414/CF-223912-a06** | **Inhibited mycelium** | **Palmarumycin C15, Preussomerin F** | **6.5** | **8.1** | **10.1** | **9.7** | **14.5** | **-95.32** |
| **CF-246868-a06-MO001-EC01** | **Axenic** | **Palmarumycin C15, Preussomerin L** | **0** | **7.1** | **6.7** | **0** | **7.5** | **-86.86** |
| **CF-246868-a06-MO002-EC01** | **Axenic** | **Palmarumycin C15, Preussomerin L** | **0** | **7.5** | **7.4** | **0** | **7.8** | **-83.65** |
| **CBS 102414/CF-246868-a05** | **Inhibition zone** | **Palmarumycin C15, Preussomerin L** | **6.8** | **7.1** | **6.6** | **0** | **8.8** | **-95.46** |
| **CBS 102414/CF-246868-a05** | **Inhibited mycelium** | **Palmarumycin C15, Preussomerin L** | **5.8** | **8.6** | **6.8** | **0** | **9.1** | **-96.74** |
| **CF-255912-a03-MO001-EC01** | **Axenic** |  | **0** | **0** | **0** | **0** | **0** | **7.09** |
| **CF-255912-a03-MO002-EC01** | **Axenic** |  | **0** | **0** | **0** | **0** | **0** | **19.19** |
| **CBS 102414/CF-255912-a02** | **Inhibition zone** |  | **0** | **0** | **0** | **0** | **0** | **46.77** |
| **CBS 102414/CF-255912-a02** | **Inhibited mycelium** |  | **0** | **0** | **0** | **0** | **0** | **34.28** |
| **CF-257866-a04-MO001-EC01** | **Axenic** | **7-Chloro-6-methoxymellein** | **0** | **0** | **0** | **0** | **0** | **-17.85** |
| **CF-257866-a04-MO002-EC01** | **Axenic** | **7-Chloro-6-methoxymellein** | **0** | **0** | **0** | **0** | **0** | **-2.22** |
| **CBS 102414/CF-257866-a03** | **Inhibition zone** |  | **0** | **0** | **0** | **0** | **0** | **56.15** |
| **CBS 102414/CF-257866-a03** | **Inhibited mycelium** |  | **0** | **0** | **7.8** | **0** | **0** | **32.59** |
| **CF-258646-a03-MO001-EC01** | **Axenic** |  | **0** | **0** | **0** | **0** | **0** | **9.26** |
| **CF-258646-a03-MO002-EC01** | **Axenic** |  | **0** | **0** | **0** | **0** | **0** | **-2.95** |
| **CBS 102414/CF-258646-a02** | **Inhibition zone** |  | **0** | **0** | **5.7** | **0** | **0** | **2.22** |
| **CBS 102414/CF-258646-a02** | **Inhibited mycelium** |  | **4.9** | **7.1** | **6.2** | **0** | **0** | **-78.81** |
| **CF-258778-a04-MO001-EC01** | **Axenic** | **Palmarumycin C15, Preussomerin B** | **0** | **0** | **0** | **0** | **0** | **-99.79** |
| **CF-258778-a04-MO002-EC01** | **Axenic** | **Palmarumycin C15, Preussomerin B** | **0** | **0** | **0** | **0** | **0** | **-96.48** |
| **CBS 102414/CF-258778-a03** | **Inhibition zone** | **Preussomerin B, F** | **6.1** | **5.8** | **6.2** | **0** | **7.2** | **-98.44** |
| **CBS 102414/CF-258778-a03** | **Inhibited mycelium** | **Preussomerin B, F** | **5.2** | **7.2** | **6.4** | **0** | **0** | **-98.86** |
| **CF-258809-a04-MO001-EC01** | **Axenic** |  | **0** | **0** | **0** | **0** | **0** | **-26.44** |
| **CF-258809-a04-MO002-EC01** | **Axenic** |  | **0** | **0** | **0** | **0** | **0** | **4.19** |
| **CBS 102414/CF-258809-a03** | **Inhibition zone** |  | **0** | **0** | **0** | **0** | **0** | **43.79** |
| **CBS 102414/CF-258809-a03** | **Inhibited mycelium** |  | **0** | **0** | **0** | **0** | **0** | **78.00** |
| **CF-260282-a06-MO001-EC01** | **Axenic** |  | **0** | **0** | **0** | **0** | **0** | **10.19** |
| **CF-260282-a06-MO002-EC01** | **Axenic** |  | **0** | **0** | **0** | **0** | **0** | **11.54** |
| **CBS 102414/CF-260282-a04** | **Inhibition zone** | **Preussomerin B, F** | **6.3** | **6.8** | **0** | **0** | **0** | **-85.24** |
| **CBS 102414/CF-260282-a04** | **Inhibited mycelium** | **Preussomerin B, F** | **6.1** | **7.2** | **0** | **0** | **7.9** | **-96.59** |
| **CF-260308-a04-MO001-EC01** | **Axenic** |  | **0** | **0** | **0** | **0** | **0** | **-10.81** |
| **CF-260308-a04-MO002-EC01** | **Axenic** |  | **0** | **0** | **0** | **0** | **0** | **3.88** |
| **CBS 102414/CF-260308-a02** | **Inhibition zone** |  | **6.4** | **5.7** | **6.2** | **0** | **0** | **-99.01** |
| **CBS 102414/CF-260308-a02** | **Inhibited mycelium** |  | **6.1** | **7.1** | **6.5** | **0** | **0** | **-89.21** |
| **CF-262323-a04-MO001-EC01** | **Axenic** |  | **0** | **0** | **0** | **0** | **0** | **-30.41** |
| **CF-262323-a04-MO002-EC01** | **Axenic** |  | **0** | **0** | **0** | **0** | **0** | **13.36** |
| **CBS 102414/CF-262323-a03** | **Inhibition zone** |  | **6.8** | **6.3** | **6.8** | **0** | **0** | **1.92** |
| **CBS 102414/CF-262323-a03** | **Inhibited mycelium** |  | **0** | **0** | **6.9** | **0** | **0** | **-11.57** |
| **CF-267373-a04-MO001-EC01** | **Axenic** |  | **0** | **0** | **0** | **0** | **0** | **39.06** |
| **CF-267373-a04-MO002-EC01** | **Axenic** |  | **0** | **0** | **0** | **0** | **0** | **39.95** |
| **CBS 102414/CF-267373-a03** | **Inhibition zone** |  | **6.8** | **0** | **7.4** | **0** | **0** | **-21.22** |
| **CBS 102414/CF-267373-a03** | **Inhibited mycelium** |  | **6.3** | **7.2** | **6** | **0** | **0** | **-94.46** |
| **CF-268679-a03-MO001-EC01** | **Axenic** |  | **5.7** | **0** | **0** | **7.6** | **0** | **48.73** |
| **CF-268679-a03-MO002-EC01** | **Axenic** |  | **0** | **0** | **0** | **6.1** | **0** | **43.13** |
| **CBS 102414/CF-268679-a02** | **Inhibition zone** |  | **0** | **0** | **0** | **0** | **0** | **94.75** |
| **CBS 102414/CF-268679-a02** | **Inhibited mycelium** |  | **0** | **0** | **0** | **0** | **0** | **95.74** |
| **CF-268787-a04-MO001-EC01** | **Axenic** |  | **0** | **0** | **0** | **0** | **0** | **11.32** |
| **CF-268787-a04-MO002-EC01** | **Axenic** |  | **0** | **0** | **0** | **0** | **0** | **28.50** |
| **CBS 102414/CF-268787-a03** | **Inhibition zone** |  | **6.3** | **7.6** | **6.3** | **0** | **8.7** | **-98.58** |
| **CBS 102414/CF-268787-a03** | **Inhibited mycelium** |  | **6.6** | **6.9** | **5.8** | **0** | **0** | **-98.86** |
| **CF-268841-a04-MO001-EC01** | **Axenic** | **Globosuxanthone A** | **0** | **0** | **0** | **0** | **0** | **-48.85** |
| **CF-268841-a04-MO002-EC01** | **Axenic** | **Globosuxanthone A** | **0** | **0** | **0** | **0** | **0** | **-21.63** |
| **CBS 102414/CF-268841-a03** | **Inhibition zone** | **Globosuxanthone A** | **6.5** | **6.8** | **6.9** | **0** | **0** | **-99.57** |
| **CBS 102414/CF-268841-a03** | **Inhibited mycelium** | **Globosuxanthone A** | **7** | **6.7** | **0** | **0** | **0** | **-99.57** |
| **CF-269659-a05-MO001-EC01** | **Axenic** | **Palmarumycin C15, Preussomerin B, Preussomerin L** | **0** | **0** | **0** | **0** | **0** | **-89.44** |
| **CF-269659-a05-MO002-EC01** | **Axenic** | **Palmarumycin C15, Preussomerin B, Preussomerin L** | **0** | **0** | **6.9** | **0** | **0** | **-95.17** |
| **CBS 102414/CF-269659-a04** | **Inhibition zone** | **Palmarumycin C15, Preussomerin B** | **0** | **0** | **0** | **0** | **0** | **-69.06** |
| **CBS 102414/CF-269659-a04** | **Inhibited mycelium** | **Preussomerin B, F** | **6.4** | **8** | **8.2** | **0** | **0** | **-97.73** |
| **CF-269692-a05-MO001-EC01** | **Axenic** |  | **0** | **0** | **5.7** | **0** | **0** | **-93.89** |
| **CF-269692-a05-MO002-EC01** | **Axenic** |  | **0** | **0** | **0** | **0** | **0** | **36.77** |
| **CBS 102414/CF-269692-a04** | **Inhibition zone** |  | **5.5** | **6.9** | **6.6** | **0** | **0** | **-99.15** |
| **CBS 102414/CF-269692-a04** | **Inhibited mycelium** |  | **5.8** | **6.7** | **5.5** | **0** | **0** | **-68.06** |
| **CF-277042-a05-MO001-EC01** | **Axenic** |  | **0** | **0** | **0** | **0** | **0** | **40.46** |
| **CF-277042-a05-MO002-EC01** | **Axenic** |  | **0** | **0** | **0** | **0** | **0** | **23.92** |
| **CBS 102414/CF-277042-a04** | **Inhibition zone** |  | **0** | **0** | **0** | **0** | **0** | **14.69** |
| **CBS 102414/CF-277042-a04** | **Inhibited mycelium** |  | **0** | **0** | **0** | **0** | **0** | **31.58** |
| **CF-284867-a05-MO001-EC01** | **Axenic** | **Griseofulvin** | **0** | **0** | **0** | **0** | **0** | **-81.93** |
| **CF-284867-a05-MO002-EC01** | **Axenic** | **Griseofulvin** | **0** | **0** | **0** | **0** | **0** | **-87.91** |
| **CBS 102414/CF-284867-a02** | **Inhibition zone** | **Griseofulvin** | **0** | **0** | **7.2** | **0** | **0** | **17.63** |
| **CBS 102414/CF-284867-a02** | **Inhibited mycelium** | **Griseofulvin** | **0** | **0** | **0** | **0** | **0** | **5.63** |
| **CF-285353-a07-MO001-EC01** | **Axenic** |  | **0** | **0** | **0** | **0** | **0** | **28.75** |
| **CF-285353-a07-MO002-EC01** | **Axenic** |  | **0** | **0** | **0** | **0** | **0** | **29.13** |
| **CBS 102414/CF-285353-a06** | **Inhibition zone** |  | **0** | **0** | **6.1** | **0** | **0** | **95.26** |
| **CBS 102414/CF-285353-a06** | **Inhibited mycelium** |  | **0** | **0** | **0** | **0** | **0** | **25.93** |
| **CF-288225-a03-MO001-EC01** | **Axenic** |  | **0** | **0** | **0** | **0** | **11.9** | **60.43** |
| **CF-288225-a03-MO002-EC01** | **Axenic** |  | **0** | **0** | **0** | **0** | **0** | **47.96** |
| **CBS 102414/CF-288225-a02** | **Inhibition zone** |  | **0** | **7.3** | **6.6** | **0** | **0** | **-24.59** |
| **CBS 102414/CF-288225-a02** | **Inhibited mycelium** |  | **5.6** | **7** | **6.8** | **0** | **0** | **-94.37** |
